# Genetic fine mapping of systemic lupus erythematosus MHC associations in Europeans and African Americans

**DOI:** 10.1093/hmg/ddy280

**Published:** 2018-07-31

**Authors:** Ken B Hanscombe, David L Morris, Janelle A Noble, Alexander T Dilthey, Philip Tombleson, Kenneth M Kaufman, Mary Comeau, Carl D Langefeld, Marta E Alarcon-Riquelme, Patrick M Gaffney, Chaim O Jacob, Kathy L Sivils, Betty P Tsao, Graciela S Alarcon, Elizabeth E Brown, Jennifer Croker, Jeff Edberg, Gary Gilkeson, Judith A James, Diane L Kamen, Jennifer A Kelly, Joseph McCune, Joan T Merrill, Michelle Petri, Rosalind Ramsey-Goldman, John D Reveille, Jane E Salmon, Hal Scofield, Tammy Utset, Daniel J Wallace, Michael H Weisman, Robert P Kimberly, John B Harley, Cathryn M Lewis, Lindsey A Criswell, Timothy J Vyse

**Affiliations:** 1 Department of Medical and Molecular Genetics, King’s College London, London, UK; 2 CHORI, Children’s Hospital Oakland Research Institute, Oakland, California, USA; 3 Wellcome Trust Centre for Human Genetics, University of Oxford, UK; 4 Center for Autoimmune Genomics and Etiology (CAGE), Department of Pediatrics, Cincinnati Children’s Medical Center & University of Cincinnati and the US Department of Veterans Affairs Medical Center, Cincinnati, OH, USA; 5 Center for Public Health Genomics, Wake Forest School of Medicine, Winston-Salem, NC, USA; 6 Pfizer-University of Granada-Junta de Andalucía Centre for Genomics and Oncological Research (GENYO) , Granada, Spain; 7 Unit of Chronic Inflammation, Institute of Environmental Medicine, Karolinska Institute, Sweden; 8 Arthritis & Clinical Immunology Research Program, Division of Genomics and Data Sciences, Oklahoma Medical Research Foundation, Oklahoma City, OK, USA; 9 Keck School of Medicine of USC, Los Angeles, CA, USA; 10 Department of Medicine, Medical University of South Carolina, Charleston, SC, USA; 11 Division of Clinical Immunology and Rheumatology, University of Alabama at Birmingham, Birmingham, AL, USA; 12 Department of Pathology, University of Alabama at Birmingham, Birmingham, AL, USA; 13 Center for Clinical and Translational Science, University of Alabama at Birmingham, Birmingham, AL, USA; 14 Division of Rheumatology, Medical University of South Carolina, Charleston, SC, USA; 15 Michigan Medicine Rheumatology Clinic,Taubman Center Floor 3 Reception A, 1500 E Medical Center Dr SPC 5358, Ann Arbor, MI, USA; 16 Oklahoma Medical Research Foundation,825 N.E. 13th Street, Oklahoma City, OK, USA; 17 Division of Rheumatology, Department of Medicine, Johns Hopkins University School of Medicine, Baltimore, Maryland, USA; 18 Feinberg School of Medicine,McGaw Pavilion Suite M-300, 240 E Huron, Chicago, IL, USA; 19 Department of Internal Medicine, The University of Texas, Fannin, MSB, Houston, TX, USA; 20 Division of Rheumatology, Hospital for Special Surgery-Weill Cornell Medicine, New York, NY, USA; 21 Oklahoma Clinical and Translational Science Institute,University of Oklahoma Health Sciences Center, 920 NE Stanton L. Young, Oklahoma City, OK, USA; 22 University of Chicago Pritzker School of Medicine, Chicago, IL, USA; 23 Division of Rheumatology, Cedars Sinai Medical Center, Los Angeles, CA, USA; 24 MRC Social, Genetic and Developmental Psychiatry Centre, Institute of Psychiatry, Psychology & Neuroscience, King’s College London, London, UK; 25 Rosalind Russell / Ephraim P Engleman Rheumatology Research Center, Division of Rheumatology, UCSF School of Medicine, San Francisco, CA, USA

## Abstract

Genetic variation within the major histocompatibility complex (MHC) contributes substantial risk for systemic lupus erythematosus, but high gene density, extreme polymorphism and extensive linkage disequilibrium (LD) have made fine mapping challenging. To address the problem, we compared two association techniques in two ancestrally diverse populations, African Americans (AAs) and Europeans (EURs). We observed a greater number of Human Leucocyte Antigen (HLA) alleles in AA consistent with the elevated level of recombination in this population. In EUR we observed 50 different *A—C—B—DRB1—DQA—DQB* multilocus haplotype sequences per hundred individuals; in the AA sample, these multilocus haplotypes were twice as common compared to Europeans. We also observed a strong narrow class II signal in AA as opposed to the long-range LD observed in EUR that includes class I alleles. We performed a Bayesian model choice of the classical HLA alleles and a frequentist analysis that combined both single nucleotide polymorphisms (SNPs) and classical HLA alleles. Both analyses converged on a similar subset of risk HLA alleles: in EUR HLA– B^*^08:01 + B^*^18:01 + (DRB1^*^15:01 frequentist only) + DQA^*^01:02 + DQB^*^02:01 + DRB3^*^02 and in AA HLA–C^*^17:01 + B^*^08:01 + DRB1^*^15:03 + (DQA^*^01:02 frequentist only) + DQA^*^02:01 + DQA^*^05:01+ DQA^*^05:05 + DQB^*^03:19 + DQB^*^02:02. We observed two additional independent SNP associations in both populations: EUR rs146903072 and rs501480; AA rs389883 and rs114118665. The DR2 serotype was best explained by DRB1^*^15:03 + DQA^*^01:02 in AA and by DRB1^*^15:01 + DQA^*^01:02 in EUR. The DR3 serotype was best explained by DQA^*^05:01 in AA and by DQB^*^02:01 in EUR. Despite some differences in underlying HLA allele risk models in EUR and AA, SNP signals across the extended MHC showed remarkable similarity and significant concordance in direction of effect for risk-associated variants.

## Introduction

Systemic lupus erythematosus (SLE) is a highly complex disease, with occurrence heavily influenced by genetics (heritability = 44%; (1)). SLE incidence varies markedly across populations, with Europeans (EURs) showing 3–4-fold lower prevalence compared with individuals of African or Asian ancestry (2,3). Genome-wide association studies (GWAS) indicate a strong genetic signal arising from the major histocompatibility complex (MHC) in all populations studied (4–6). The association signals in the MHC have been studied in EURs (7) and East Asians (8–10). In EURs, the strength of the MHC signal seen in GWAS is driven by multiple separate genetic factors. Unravelling these different effects is hampered by extensive linkage disequilibrium (LD). Two SLE-associated haplotypes that exhibit extended LD have been described in EURs: the haplotypes include the *HLA-DRB1* alleles, HLA-DRB1^*^03:01 and HLA-DRB1^*^15:01. These two haplotypes are also associated with other autoimmune diseases (11,12) and are often referred to by their tagging *HLA-DRB1* alleles, with haplotypes containing DRB1^*^03 alleles being the ‘DR3’ serotype; haplotypes containing DRB1^*^15 or DRB1^*^16 alleles comprise the ‘DR2’ serotype. The actual causal alleles at the MHC in EURs are unknown, a somewhat surprising situation given the comparatively, in complex trait terms, large relative risk of at least two conveyed by MHC alleles. The limitation has principally been the extended LD at the MHC. In East Asian SLE the MHC risk is also strong, but may be slightly simpler than in EURs, the predominant risk arising from the extended haplotypes including HLA-DRB1^*^15:02 in LD with DQA1^*^01 and DQB1^*^05 or ^*^06 alleles (9,10). Investigation of the MHC associations in African Americans (AAs) has only previously been studied intensively in small cohorts and using limited genotyping (13) or as part of a larger scan of immune-related loci using the Immunochip (14) with limited information on HLA alleles. Small studies have implicated HLA-DRB1^*^15:03-DQA1^*^01:02-DQB1^*^06:02^13^ and a modest SNP-based study did suggest that multiple MHC association signals were present (13). Population admixture is a complicating factor in the genetic analysis in AAs.

The greater prevalence of SLE in non-EUR populations rationalizes a trans-ancestral approach to fine map genetic association signals. We have previously employed this strategy at a genome-wide level (15) and we have fine-mapped individual loci identifying a single polymorphism, likely to be causal, close to the transcription start of the SLE susceptibility gene, *TNFSF4* (16). In a small SNP-based study, we examined the pattern of association with SLE at the MHC in northern and southern EUR cohorts and in a Filipino population (10). Aligning the patterns of association suggested some similarity but revealed differences in LD around these association signals. These results suggest that trans-ancestral fine mapping strategy at the MHC is of value. A recent trans-ancestral study using the Immunochip (14) did look at HLA and SNP associations in the MHC but was not focused on the MHC and the analysis used a simple stepwise approach with a generous level of statistical significance for inclusion. The Immunochip study was also limited by a small number of AA ancestry samples in the reference data used for HLA imputation.

We have genotyped 1494 SLE cases and 5908 controls of AA ancestry for genetic markers within the MHC, as part of a GWAS. 308 AA subjects were also genotyped for classical class II HLA alleles and included in the reference data for HLA imputation. These data were compared to an equivalent analysis of MHC data from a recent GWAS in a EUR population (4). We performed two parallel analyses to determine the model of association for HLA alleles: 1) an analysis guided by the a priori view of causality in the Class II region and 2) a fully Bayesian model choice. The classical approach started from an assumption of association at class II loci and was motivated by the observed association signal in this area combined with the relatively short-range LD in the AA population. The Bayesian approach used Reversible Jump Markov Chain Monte Carlo (RJMCMC) simulation to search over all possible HLA models of association, with defined priors (see Materials and Methods) for genetic risk effects (odds ratios) and model size (the number of causal variants). We found that our two analyses strategies converged to very similar results for association in the HLA region.

## Results

We analysed genetic data across the MHC in AA and EUR for association with SLE. The EUR data were taken from a previously published GWAS (4) comprising 4036 cases and 6959 controls. Post quality control (QC) (see Materials and Methods) there were 6079 SNPs in the MHC (Chr6, 26–34 Mb). 1494 cases and 5908 controls of AA ancestry, genotyped as part of a GWAS (unpublished), passed QC as did 4222 SNPs within the MHC.

We generated a new reference panel of HLA-typed individuals in a subset of the AA data. A total of 308 subjects were genotyped for classical class II HLA alleles (HLA^*^DQA, HLA^*^DQB and HLA^*^DRB1) by targeted sequencing of exons 2 and 3 (*HLA-DQA* and *HLA-DQB*) and exon 2 (*HLA-DRB1*) (17). These were added to the database of reference HLA genotypes for HLA imputation with the software HLA^*^IMPV2 (18). We imputed HLA alleles in each populations’ data (see Materials and Methods) using HLA^*^IMPV2 and also imputed amino acid data (see Materials and Methods).

### Overall patterns of MHC genetic association

**Figure 1:**
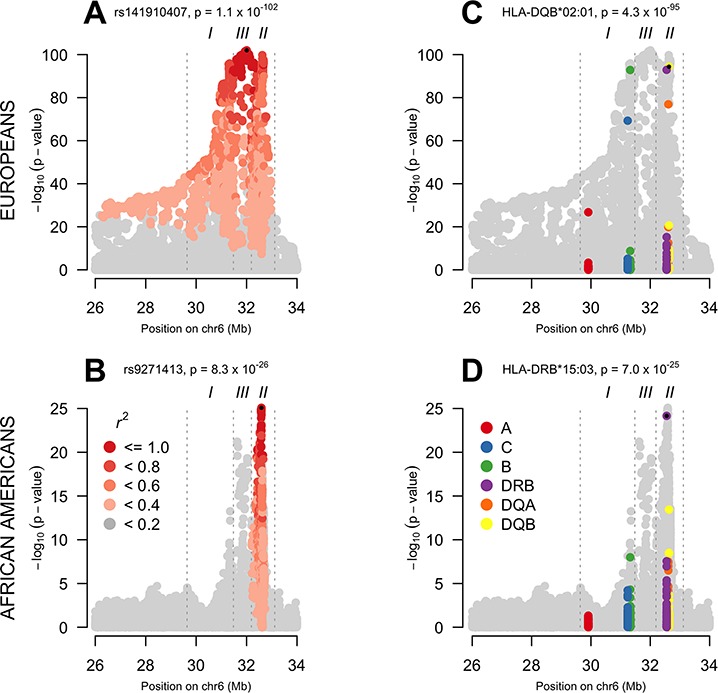
**Association signal across the extended MHC region in EUR and AA data.** In each panel, the title contains the most significant genetic marker and its *P*-value. A small black dot indicates the most significant marker. (**A**) LD with the most significant SNP in EUR. In EUR, a high level of LD exists across the entire extended MHC. (**B**) LD with the most significant SNP in AA. In AA, LD with the most significant SNP is restricted to a single peak in class II. (**C**) Association signal in class I and class II classical HLA alleles in EUR. The classical HLA alleles reflect the signal of the greyed-out SNPs from which they were imputed. (**D**) Association signal in classes I and II classical HLA alleles in AA.

We first investigated the single-marker association signals for SNPs and HLA alleles across the MHC in both populations (AA and EUR). EURs show extensive LD encompassing the entire extended MHC; in the AA data, the correlation is limited to a narrow peak in the HLA class II region (Fig. 1). The most significant HLA signal in both EUR (HLA-DQB^*^02:01, *P*-value = 4.3 × 10^−95^) and AA (HLA-DRB1^*^15:03, *P*-value = 7.0 × 10^−25^) is a class II gene. Each of these HLA alleles tags well-known associated haplotypes: HLA-DRB1^*^03:01—HLA-DQA1^*^05:01—HLA-DQB1^*^02:01 (DR3) in EURs and HLA-DRB1^*^15:03—HLA-DQA1^*^01:02—HLA-DQB1^*^06:02 (DR2) in Africans. The most associated SNP in the EUR data is tagging DR3 (R^2^ = 0.65 with HLA-DRB1^*^0301 and R^2^ = 0.74 with HLA-B^*^0801) while the most associated AA SNP is tagging DR2 (R^2^ with HLA-DRB1^*^15:03 = 0.78 and R^2^ = 0.7 with HLA-DQB1^*^06:02). The two populations show a similar genetic association signal overall as shown by the concordance in SNP associations in Supplementary Material, Fig. S1.

**Table 1:** AA association results for HLA alleles class II led stepwise regression

	Conditional results (multiple regression)			Single-marker results		
ALLELE	Odds Ratio	95% C.I.	P	Odds Ratio	95% C.I.	P
HLA-C^*^17:01	1.42	1.21–1.65	7.40E-06	1.25	1.08–1.42	2.64E-03
HLA-B^*^08:01	1.75	1.41–2.17	1.01E-07	1.93	1.59–2.35	9.74E-12
HLA-DRB1^*^15:03	2.03	1.73–2.37	1.47E-18	1.86	1.65–2.09	9.72E-26
HLA-DQA^*^01:02	1.13	0.98–1.29	8.64E-02	1.23	1.11–1.36	5.22E-06
HLA-DQA^*^02:01	0.31	0.19–0.49	1.14E-06	0.90	0.78–1.02	1.24E-01
HLA-DQA^*^05:01	1.46	1.27–1.67	4.11E-09	1.38	1.22–1.54	9.52E-09
HLA-DQA^*^05:05	0.17	0.07–0.35	4.74E-06	0.19	0.09–0.37	1.47E-06
HLA-DQB^*^03:19	1.65	1.38–1.96	2.01E-09	1.62	1.38–1.89	9.85E-10
HLA-DQB^*^02:02	6.23	3.53–11.0	1.50E-10	1.11	0.92–1.31	2.63E-01

Alleles in LD with HLA-DRB1^*^03:01 (DR3) are coloured in red, and alleles in LD with HLA-DRB1^*^15 (DR2) are coloured in purple. AIC = 7119, BIC = 7195.

### Fine mapping the class II signal

**Figure 2:**
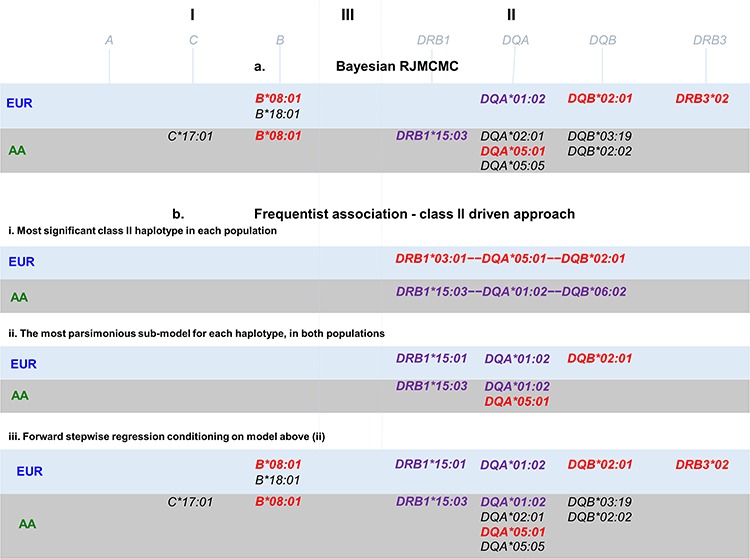
**Models of association across HLA alleles.** (**A**) Bayesian model choice fit using RJMCMC. (**B-i**) Most associated class II haplotypes, (**B-ii**) models with lowest AIC and BIC comprising classes I and II alleles. (**B-iii**) Stepwise regression starting from the alleles in B-ii. Alleles in LD with HLA-DRB1^*^03:01 (DR3) are coloured in red, and alleles in LD with HLA-DRB1^*^15 (DR2) are coloured in purple.

We were interested to determine the most likely HLA alleles that explained the class II signal in the AA and EUR data in Figure 1. Therefore, we conducted a haplotype analyses followed by a model selection analysis (see Materials and Methods and Supplementary Material 1) in both populations. This approach began with a focus on the two most associated class II DR-DQ haplotypes in each population representing DR2 and DR3 (Fig. 2B-i).

**Table 2:** EUR association results for HLA alleles in models of association from class II led stepwise regression

	Conditional results (multiple regression)			Single marker results		
	Stepwise regression					
ALLELE	OR	95% C.I.	P	OR	**95% C.I.**	**P**
HLA-B^*^08:01	1.63	1.45–1.83	1.13E-14	2.41	2.22–2.60	1.47E-93
HLA-B^*^18:01	1.40	1.24–1.58	1.64E-07	1.28	1.14–1.44	3.03E-05
HLA-DRB1^*^15:01	1.20	1.04–1.37	7.55E-03	1.32	1.22–1.43	4.53E-11
HLA-DQA^*^01:02	1.27	1.13–1.42	6.74E-05	1.27	1.17–1.37	6.36E-11
HLA-DQB^*^02:01	1.84	1.63–2.07	1.29E-24	2.32	2.14–2.50	4.34E-95
HLA-DRB3^*^02	0.76	0.70–0.82	1.01E-10	0.70	0.65–0.76	5.46E-21

Alleles in LD with HLA-DRB1^*^03:01 (DR3) are coloured in red, and alleles in LD with HLA-DRB1^*^15 (DR2) are coloured in purple. AIC = 13319, BIC = 13392.

In AA: DRB1^*^15:03—DQA^*^01:02—DQB^*^06:02 (*P*-value =7.18 ×10^−22^, OR = 1.74) and DRB1^*^03:01—DQA^*^05:01—DQB^*^02:01 (*P*-value = 3.42 × 10^−03^, OR = 1.27).

In EUR: DRB1^*^15:01—DQA^*^01:02—DQB^*^06:02 (*P*-value =8.23 ×10^−10^, OR = 1.30) and DRB1^*^03:01—DQA^*^05:01—DQB^*^02:01 (*P*-value = 2.58 × 10^−95^, OR = 2.32).

We found that DR2 was best explained by DRB1^*^15:03 +DQA^*^01:02 in AA and by DRB1^*^15:01 + DQA^*^01:02 in EUR, while DR3 was best explained by DQA^*^05:01 in AA and by DQB^*^02:01 in EUR. These alleles are noted in Figure 2B-ii.

### Stepwise regression on HLA alleles

Having determined the most likely explanation for the class II association peak in each population, we then conditioned on these models to find additional independently associated HLA alleles. We ran a forward stepwise regression on all HLA alleles starting from the class II HLA alleles just discussed (see Supplementary Material 2). This biased approach to stepwise regression, reassuringly, resulted (Fig. 2B-iii) in mainly the same HLA alleles as a fully Bayesian agnostic analysis that searched over all HLA alleles in classes I and II (see Materials and Methods, Fig. 2B-i and Supplementary Material 3). The exception being the models from this stepwise approach starting from class II includes both the HLA-DQA^*^01:02 and the HLA-DRB1^*^15 alleles whereas the Bayesian model choice includes only HLA-DQA^*^01:02 in the EUR data and only HLA-DRB1^*^15:03 in the AA data (Table 1). The colour codes in Figure 2 highlight which HLA alleles lay on the DR2 and DR3 risk haplotypes discussed above. Other alleles, such as class I B^*^18:01 in EUR and C^*^17:01 in AA, for example, are associated in addition to and independently of the risk haplotypes.

### Associations conditional on the HLA alleles

**Figure 3:**
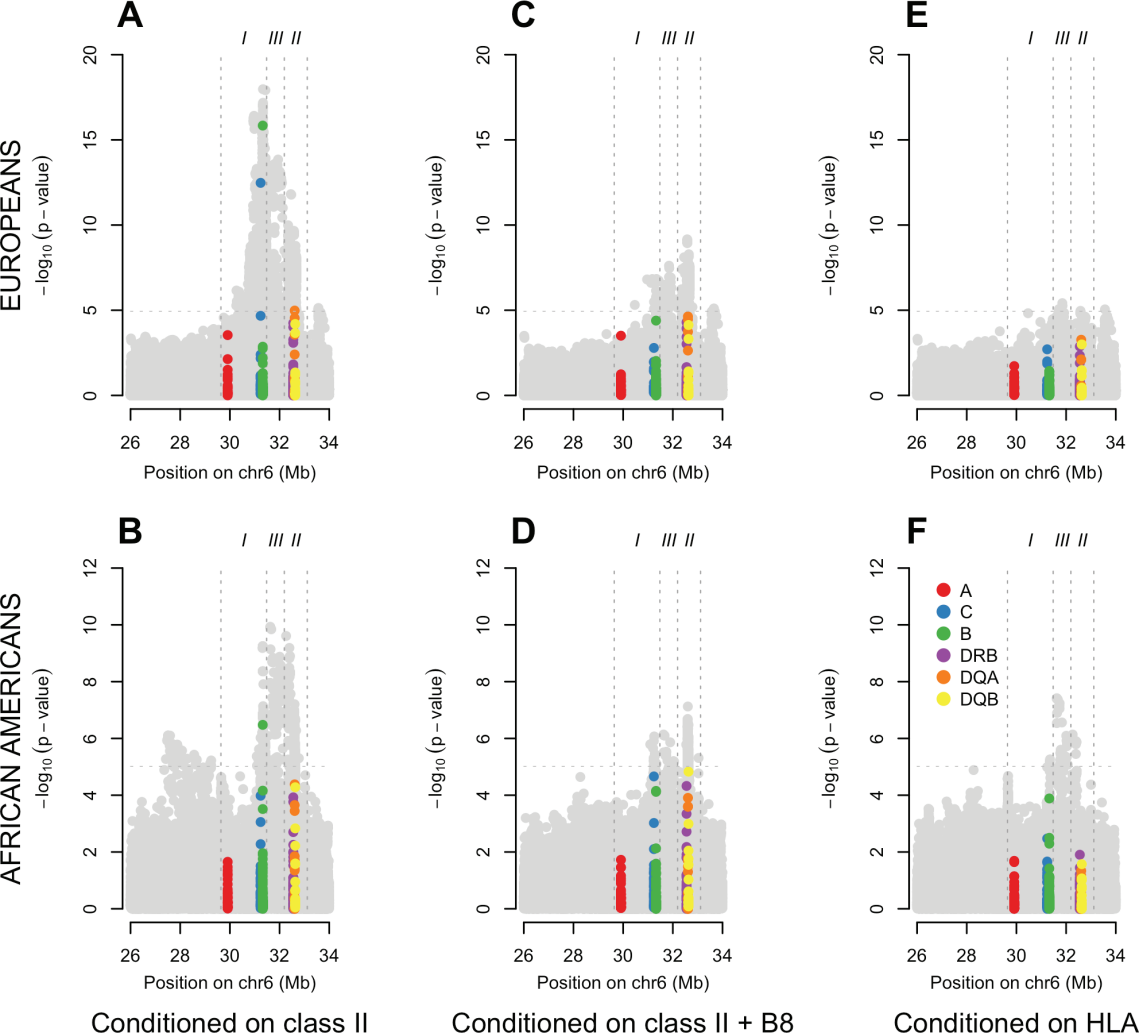
**Class II informed conditional analyses.** EUR (**A**) and AA (**B**) SNP and classical HLA allele association signals after conditioning on the best class II model (EUR: HLA-DRB1^*^15:01 + HLA-DQA^*^01:02 + HLA-DQB^*^02:01; AA: HLA-DRB1^*^15:03 + HLA-DQA^*^01:02 + HLA-DQA^*^05:01). EUR (**C**) and AA (**D**) SNP and classical HLA allele association signals after conditioning on the best class II + I model (EUR: HLA-DRB1^*^15:01 + HLA-DQA^*^01:02 + HLA-DQB^*^02:01 + HLA-B^*^08:01; AA: HLA-DRB1^*^15:03 + HLA-DQA^*^01:02 + HLA-DQA^*^05:01 + HLA-B^*^08:01). EUR (**E**) and AA (**F**) SNP and classical HLA allele association signals after conditioning on the best overall HLA model (EUR: HLA-DRB1^*^15:01 + HLA-DQA^*^01:02 + HLA-DQB^*^02:01 + HLA-DR3^*^02:01 + HLA-B^*^08:01 + HLA-B^*^18:01; AA: HLA-DRB1^*^15:03 + HLA-DQA^*^01:02 + HLA-DQA^*^02:01 + HLA-DQA^*^05:01 HLA-DQA^*^05:05 + HLA-DQB^*^03:19 + HLA-DQB^*^02:02 + HLA-B^*^08:01 + HLA-C^*^07:01).

To search for SNP associations in addition to and independent of HLA alleles, and to understand the independent regional HLA associations, we ran stepwise regression conditional on various sets of HLA alleles. Figure 3 displays association results in a sequential fashion conditional on various sets of associated HLA alleles. Figure 3A and B show the results after conditioning on the best model of association at class II; Figure 3C and D are conditioning on the best model of association for class II including the extended ancestral MHC DR3 haplotype (see Supplementary Material 4), which is effectively the class I signal from HLA-B8. Figure 3E and F show residual association after removing the signals from the best model of all HLA alleles. After conditioning on the top HLA class II association signals in each cohort, it is apparent that both cohorts show evidence of additional association signals close to the junction of MHC classes I and III regions. Class I *HLA-B8* (or variants highly correlated with it) makes a major contribution to both of these association signals, as the association spike is markedly diminished when conditional on HLA-B^*^08:01. Interestingly, when conditioning on the best overall model for HLA association there is limited evidence for further signals in the EUR cohort; however, there remains clear evidence for further association in the AA cohort in the class III region (Fig. 3F).

The stepwise regression on SNPs only using each population’s data and conditioning on the respective HLA alleles in Figure 2iii returned two SNPs in the EUR data (rs146903072: *P*-value = 3.93 × 10^−06^, OR = 1.82 95% CI 1.39–2.37, 31,847,180bp, intergenic *SLC44A4* – *EHMT2*; rs501480: *P*-value = 9.84 ×10^−06^, OR = 1.15 95% CI 1.08–1.22, 33,563,946 bp, intergenic *GGNBP1* – *LINC00336*) and two SNPs in the AA data (rs389883:*P*-value = 4.37 × 10^−08^, OR = 1.76 95% CI 1.31–1.76, 31947460 bp, intron *STK19*; rs114118665: *P*-value = 5.76 × 10^−06^, OR = 2.37 95% CI 1.56–3.60, 31342005 bp, intergenic *HLA-B* – *MICA*). The two associated SNPs in the AA data are not in LD with the two associated SNPs in the EUR data (R^2^ < 0.01 in all parings, in both populations). We found no evidence of association for the AA SNPs in the EUR data (as single markers of conditional on the HLA) and vice versa.

### The HLA-DQ heterodimer risk profile

As the cell surface HLA-DQ molecule is a heterodimer with variation in both its alpha (coded *DQA*) and beta (coded *DQB*) chains, we explored the hypothesis that a combination of *DQA* and *DQB* alleles would be a better model fit than including the alleles as independently associated. We found no evidence (see Materials and Methods) in favour of an interaction model between any pair of *DQA* and *DQB* alleles. Furthermore, we found no specific combination of DQA and DQB alleles that fit the data better than simple additive models. This suggests that the effects of DQA and DQB alleles are independent.

### Two-digit DRB1^*^15 association and amino acid data

We looked closely at the association signals for HLA alleles nested within the two-digit HLA-DRB1^*^15 group, as these alleles are consistently associated with SLE across major populations yet differ in frequency and in the most associated allele. The DRB1^*^15:03 allele is the most associated DRB1^*^15 allele in the AA cohort (*P*-value = 1 × 10^−25^, OR = 1.86 95% C.I. =1.66–2.09); however we did observe DRB1^*^15:01 (frequency = 3.3%) and DRB1^*^15:02 (0.3%) alleles with association *P*-values of 0.03 and 0.46, respectively, and effect size estimates of 1.32 (95% C.I. 1.03–1.69) and 1.50 (95% C.I. 0.50–4.46). In the EUR data where DRB1^*^15:01 is the most associated DRB1^*^15 allele (*P*-value = 4.53 × 10^−11^, OR = 1.32 95% C.I. =1.22–1.43), we also observe DRB1^*^15:02 (frequency = 0.8%) but with no evidence (*P*-value = 1.86 × 10^−01^, OR = 0.81 95% C.I. =0.59–1.12) for association. DRB1^*^15:02 has been found to be associated in East Asians (9), DRB1^*^15:01 has also been found to be associated in this population (19).

We tested a one-parameter two-digit DRB1^*^15 allele model against a three-parameter (a separate odds ratio for each allele: DRB1^*^15:01 + DRB1^*^15:02 + DRB1^*^15:03) model in the AA data. We did find weak evidence (*P*-value = 0.02) to reject the two-digit model using a likelihood ratio test; however the Bayesian Information Criterion (BIC) favoured the two-digit model (difference in BIC = 10.37). This has some biological significance as the three HLA alleles share the same amino acid residue at position 71 (A) and no other *HLA-DRB1* allele amongst those imputed in the AA dataset codes for this residue at this position. The two-digit model of association is therefore equivalent to an amino acid residue association.

### Comparison of HLA, amino acid and SNP models of association

An important question is whether the association signal across the MHC can be best explained by SNPs, HLA alleles or amino acid residues. To explore this we compared our results for HLA association to those obtained by stepwise regression analyses on amino acid and SNP data (Table 3). In both populations’ analyses we found that the amino acid models were a poorer fit than HLA alleles, as judged by the Akaike Information Criterion (AIC) or BIC. In the AA data, the HLA model was the best overall fit. In the EUR data, the SNP model was the best fit. The SNP model in the AA data is likely not tagging all the SLE-associated variation, in support of this interpretation we did find two further independent HLA associations, namely HLA^*^DQA^*^05:05 and HLA^*^DRB1^*^13:04, conditional on the four SNPs noted in Table 4. The HLA alleles tagged by the SNP models can be seen in Supplementary Material, Fig. S3, and for reference the full set of HLA frequencies and associations can be seen in Supplementary Material, Fig. S4.

**Table 3:** Association results for amino acid data from stepwise regression in the AA data

	Conditional results (multiple regression)			Single-marker results			HLA alleles specific to amino acid^**a**^
ALLELE	OR	95% C.I.	P	OR	95% C.I.	P	
DRB1 71A	2.22	1.97–2.49	2.82E-40	1.72	1.55–1.91	1.07E-24	DRB1^*^15:01, :02, :03
DQB -18A	1.63	1.48–1.80	1.19E-22	1.20	1.10–1.30	3.34E-05	DQB^*^02:01, :02, :03, 03:01, :04, :09, ^*^03:19, ^*^06:01
B 156D	1.57	1.40–1.76	2.20E-15	1.42	1.28–1.58	1.80E-10	B^*^08:01, ^*^37:01, :41, :42, :45, :82
DQA -13 T	0.11	0.05–0.22	2.26E-09	0.19	0.09–0.37	1.47E-06	DQA^*^05:05

a ^a^ These are the HLA alleles that are specific to the amino acid, for example, the only HLA alleles observed in our data that code for DRB-71-A are DRB1^*^15:01, DRB1^*^15:02 and DRB1^*^15:03. See Supplementary Material, Fig. S4 for HLA alleles’ frequencies in our data. AIC = 7163, BIC = 7211

**Table 4:** Association results for SNPs from stepwise regression in the AA data. AIC = 7176, BIC = 7224

	Conditional results (multiple regression)				Single-marker results		
SNP	Effect (Other) Allele	OR	95% C.I.	P	OR	95% C.I.	P
RS9271413	G (A)	2.07	1.84–2.33	4.69E-34	1.72	1.56–1.91	8.30E-26
RS9273481	C (G)	1.45	1.32–1.60	3.80E-14	1.19	1.09–1.29	6.56E-05
6:31323500:D	D (I)	1.48	1.31–1.68	7.79E-10	1.47	1.30–1.65	2.28E-10
RS115549526	T (C)	1.60	1.36–1.18	1.33E-08	2.13	1.83–2.49	6.12E-22

**Table 5:** Association results for amino acid data from stepwise regression in the EUR data

	Conditional results (multiple regression)			Single-marker results			HLA alleles specific to amino acid^**a**^
ALLELE	OR	95% C.I.	P	OR	95% C.I.	P	
DRB1 77 N	2.02	1.78–2.30	2.86E-27	2.30	2.13–2.49	1.24E-93	DRB1^*^03:01, :02
DQA 207 M	1.50	1.39–1.62	9.40E-25	1.27	1.18–1.36	6.36E-11	DQA^*^01:02
B 9D	1.66	1.47–1.87	1.90E-16	2.42	2.22–2.63	1.47E-93	B^*^08:01, ^*^39:12
DRB1 233 T	1.24	1.16–1.33	3.25E-09	0.85	0.81–0.90	7.06E-08	DRB1^*^15:01, :02, ^*^01:01, :02, :03, ^*^04:01, :02, :03, :04, :05, :07, :08, :10, ^*^07:01, ^*^08:01, :03, :04, :10, 09:01, ^*^10:01, ^*^16:01
B 30G	1.34	1.18–1.52	4.39E-06	1.28	1.14–1.44	3.45E-05	B^*^18:01, :03

a ^**a**^ **These are the HLA alleles that are specific to the amino acid. See Supplementary Material, Fig. S4 for HLA alleles’ frequencies in our data. AIC = 13335, BIC = 13409**

**Table 6:** Association results for SNPs from stepwise regression in the EUR data. AIC = 13241, BIC = 1333

	Conditional results (multiple regression)				Single-marker results		
SNP	Effect (Other) Allele	OR	95% C.I.	P	OR	95% C.I.	P
RS141910407	T (C)	1.52	1.29–1.81	1.18E-06	2.71	2.47–2.97	3.93E-99
RS9260	A (G)	1.51	1.40–1.63	6.41E-27	1.33	1.24–1.43	2.17E-15
RS9273336	T (C)	1.84	1.62–2.08	1.43E-21	2.18	2.01–2.35	1.24E-85
X6:31428746:I	I (D)	1.35	1.21–1.51	1.29E-07	2.12	1.97–2.28	6.59E-87
RS9270807	G (A)	1.25	1.17–1.34	6.00E-10	0.87	0.82–0.92	1.71E-06
RS2293861	C (T)	1.27	1.16–1.38	1.03E-07	1.48	1.36–1.61	5.78E-20
RS142903940	G (A)	1.19	1.11–1.29	3.79E-06	1.03	0.96–1.11	3.56E-01
RS501480	C (T)	1.15	1.08–1.22	7.83E-06	1.22	1.15–1.29	2.26E-11

### Autoantibody sub-phenotypes

We had data available on autoantibody levels in both populations, so we exploited this and present here novel cross-population genetic association analyses of these phenotypes.

In EUR the anti-Ro autoantibody was present in 851 of 2492 cases (34%). We found two independent significant associations with both anti-Ro and anti-La in **case-only analyses**. The most significant anti-Ro association was a class I SNP rs115924783 (31 316 080 bp; OR = 2.05 95% CI 1.76–2.39; *P*-value = 3.12 × 10^−20^) in tight LD with the classical class I allele B^*^08:01 (*r^2^* = 0.97, EUR data). The most significant anti-La association rs114469371 (32189921 bp; OR = 2.04 95% CI 1.70–2.45; *P*-value = 3.45 × 10^−14^) was less correlated with B^*^08:01 (*r^2^* = 0.60, EUR data). The secondary independent associations were rs9272780 (anti-Ro; OR = 0.62 95% CI 0.53–0.71; *P*-value = 2.26 × 10^−11^) and rs3763355 (anti-La; OR = 0.38 95% CI 0.24–0.62; *P*-value = 8.53 × 10^−06^). We also found significant SNP associations with anti-RNP (rs147810605; 32,490,331 bp; *P*-value = 5.36 × 10^−09^) and anti-dsDNA (rs116794933; 31,113,275 bp; *P*-value = 9.75 × 10^−06^). Apart from rs115924783 and rs114469371 (correlated with *HLA-B8*) none of the other SNP associations had high (*r^2^* > 0.6, EUR data) with any HLA alleles.

In AA, the anti-Ro autoantibody was present in 392 of 1200 AA cases (33%). We found some evidence of association between anti-Ro and B^*^08:01 (OR = 1.67; 95% CI = 1.16–2.42; *P*-value = 6 × 10^−03^); however B^*^08:01 has a lower frequency in AA (7.2%) compared with EUR (20.4%) controls. The only statistically significant association with anti-Ro was a ‘protective’ one and that was with DRB1^*^15:03 (OR = 0.48, 95% CI = 0.36–0.61, *P*-value = 2.13 × 10^−08^). DRB1^*^15:01 was not associated with anti-Ro in EUR (OR = 1.14, 95% CI = 0.96–1.34, *P*-value = 1.39 × 10^−01^). We did not find significant evidence of association between DRB1^*^15:03 and anti-RNP (461 cases positive) or anti-Sm (420 cases positive) (*P*-value = 1.11 × 10^−03^ and *P*-value = 1.11 ×10^−02^, respectively), although there was a trend for a risk effect (OR = 1.45; 95% C.I. = 1.67–1.79 and OR = 1.34; 95% CI = 1.06–1.69, respectively). We found no significant associations (all *P*-value > 0.01) between *DQA* or *DQB* alleles and anti-RNP or anti-Sm in the AA data.

## Discussion

Our analyses of SNP, HLA and amino acid data in the MHC in an AA and EUR population have identified the key HLA alleles that are associated with SLE together with two SNPs independently associated in both populations. We found models using HLA alleles were a better fit to the data than amino acids’ models in both the AA and EUR data. There is a similar landscape of association with two independent class II associations in both populations.

Our results for HLA associations are not the result of a single analyses using stepwise regression, as is common in analysis of a single region such as the MHC. We used two approaches: a frequentists approach to decomposing class II-associated haplotypes followed by conditional analyses and a Bayesian model choice that searches over the full model space of HLA alleles. The two approaches resulted in largely the same set of HLA alleles, while the Bayesian approach was more parsimonious by only including DQA^*^01:02 as associated in the EUR data, rather than both DRB1^*^15:01 and DQA^*^01:02. In addition, the Bayesian approach included only DRB1^*^15:03 as associated in the AA data, rather than both DRB1^*^15:03 and DQA^*^01:02. In both cases the pair of alleles is in LD (r^2^ = 0.61 and r^2^ = 0.37 in each population, respectively) and this discrepancy between the approaches demonstrates some uncertainty remains on this particular haplotype. There is some suggestion that the DRB1^*^15 two-digit allele could be the best explanation in both populations for one of the main class II haplotypes associated, and this could be further explained by a specific amino acid coding at position 71 (A) for DRB1^*^1501, DRB1^*^1502 and DRB1^*^1503.

The class II *DR3* haplotype harbouring the commonly observed SLE-associated DRB1^*^03:01 allele was best explained by DQB^*^02:01 in the EUR data and DQA^*^05:01 in the AA data. The LD between these two alleles is much lower in the AA than EUR data (r^2^ = 0.33 versus r^2^ = 0.92); thus there is more power to resolve the DR3 class II associations in AAs. Our results suggest that DQA^*^05:01 is the most likely causal HLA class II allele on this haplotype. This and the lack of extended LD, as illustrated in Figure 1, suggest that the AA data have been very useful here in fine mapping both the HLA alleles and independently associated SNPs. Both populations have evidence of additional independent associations in class I with B^*^08:01 being a consistent associated allele in the two populations.

Our findings of SNP associations independent of HLA alleles do show some consistency in the identification of two class II/III SNPs independently associated in both populations, but they also highlight some uncertainty and hence the need for more extensive sequencing at the MHC including accurate HLA typing.

We find novel *HLA-DQ* associations in the AA data (DQA^*^02:01, DQA^*^05:05 and DQB^*^02:02). There is no difference in the peptide-binding groove when replacing DQA^*^05:05 with DQA^*^05:01, which captures the DR3 signal in the AA represented by DQB^*^02:01 in the EURs. The only difference between the two products is in the 11th codon in the leader sequence [position −13; DQA^*^05:01 has GCC (alanine, non-polar and hydrophobic); DQA^*^05:05 has ACC (threonine, polar and hydrophilic). Therefore, the primary amino acid sequences of the two mature proteins are identical and should exhibit identical disease susceptibility. However, we did not sequence exon-1 of *DQA*; hence the genotyping is dependent on imputation and this, together with DQA^*^05:05 being rare in AA, leads to some uncertainty in this allele’s association.

The DQA^*^02:01 and DQB^*^02:02 alleles’ associations seem complex as these two HLA alleles are in LD with one another (R^2^ = 0.87 in the AA data); they show conditional association with a likely dominant effect for DQA^*^02:01 (OR = 0.67; 95% C.I. = 0.60–0.76; *P*-value = 1.31 × 10^−11^). It seems that DQB^*^02:02 only has a significant risk effect when conditioned on the protective (possible dominant) effect of DQA^*^02:01. We find no evidence of interaction between HLA-DQA^*^02:01 and HLA-DQB^*^02:02. Due to the two alleles being in strong LD this result could be due to omitted variable bias, which would result in each of the allele’s effect being shrunk to zero when not including both correlated variables in a model of association.

We found a significant association between B^*^08:01 and anti-Ro antibodies in a case-only analysis of the EUR data (OR = 2.03 95% CI 1.74–2.36; *P*-value = 4.02 × 10^−19^). While a class I SNP was more associated than the HLA allele, due to imputation uncertainly we cannot rule out this HLA allele as more likely causal, which would be an interesting finding in the light of the suspected role of Epstein Barr Virus (EBV) in SLE pathogenesis. *B8* binds an immune-dominant peptide from EBV EBNA antigen (20,21). This association was also seen in the AA data, but it was less significant (OR = 1.67 95% CI 1.16–2.41; *P*-value = 6.13 × 10^−03^).

In summary this study substantially extends our understanding of MHC association in SLE with the inclusion of a large-scale study of AA samples and combining with a new analysis of a large EUR dataset. We have novel HLA typing included in a subset of the AA dataset, which greatly improves imputation. We find similarity between the AA and EURs in their pattern of association across the MHC using novel and coherent fully Bayesian analyses to determine the best model of association with HLA. The AA data highlight strong evidence for association at class II independent of other loci. This has shown that comparing the results of the MHC associations in EURs and AAs assists in fine mapping these signals.

## Materials and Methods

### Samples and genotyping

EUR**s:** The EUR data were taken from a previously published GWAS (4) comprising 4036 cases and 6959 controls. Post QC (which included MAF > 0.01, differential missingness *P*-value < 5 × 10^−07^ and SNP missingness <0.05) there were 6079 SNPs in the MHC.

**AAs:** 1494 cases and 5908 controls of AA ancestry, genotyped as part of a GWAS (unpublished), passed QC. These were genotyped on the following chips: OMNI2.5 (1509 controls), Omni 1 (1494 cases and 1099 controls) and Omni Express (3300 controls).

Post QC there were 4222 SNPs within the MHC. SNPs were removed if they had greater than 2% missing data across all samples, a *P*-value <0.05 for a test of differential missing data between cases and controls, a Hardy Weinberg Equilibrium test in cases with *P*-value <10^−04^ or a Hardy Weinberg Equilibrium test in controls with *P*-value <10^−02^.

Samples were removed if their call rates <90% across good quality SNPs, had excess autosomal heterozygosity or if their genetically determined sex differed from their reported sex. Additionally, duplicate samples and first-degree relatives were removed.

A total of 308 subjects were also genotyped for classical class II HLA alleles (HLA^*^DQA, HLA^*^DQB and HLA^*^DRB1) by targeted sequencing of exons 2 and 3 (*HLA-DQA* and *HLA-DQB*) and exon 2 (*HLA-DRB1*) (17). This set included the ‘HLA reference set’ used for HLA imputation into the rest of the AA study. These were added to the database of reference HLA genotypes for HLA imputation with the software HLA^*^IMPV2^18^.

### SNP imputation 

All AA and EUR subjects were imputed up to the 1000 Genomes (Phase I integrated set V3 March 2012) density using post-QC typed SNPs using IMPUTE (22). All populations’ reference data were used for imputation in the AA and EUR data as advised by the authors of IMPUTE. We set a quality threshold of 0.7 for IMPUTE INFO score and only analysed SNPs with scores above this level.

### HLA allele imputation

HLA genotypes for *HLA-A, HLA-B, HLA-C, HLA-DQA, HLA-DQB* and *HLA-DRB1* were imputed into the AA data using HLA^*^IMP-V2 (18). The same procedure was used to impute HLA alleles in the EUR data for the classical HLA genes: *HLA-A, HLA-B, HLA-C, HLA-DQA, HLA-DQB, HLA-DRB1, HLA-DRB3, HLA-DRB4, HLA-DRB5 and HLA-DPB1*. While the same reference data was used to impute both the AA and EUR data, the additional HLA alleles imputed in the EUR data were not supported for multi-ethic samples in the HLA^*^IMP algorithm and so were not imputed in the AA data. HLA-IMP-V2 uses multi-ethnic samples as reference data including data from the 1958 British Birth Cohort, 1000 genomes subjects and additional, mainly EUR, data provided by GlaxoSmithKline. Full details of these samples can be seen in the publication paired with this software (18). Our contributed AA samples to the reference data increased the size of the AA/African background set, which was 28, 34 and 28 for *HLA-DQA1, HLA-DQB1* and *HLA-DRB1,* respectively, by over 10-fold.

For regression analyses we took the probabilistic genotypes (rather than best guess) output and converted to dosage (expected allele counts). For phasing and haplotype analyses we took the best guess genotypes.

### HLA imputation assessment

HLA^*^IMP-V2 (18) performs cross validation on all reference samples (two-thirds are used for reference and one-third, for validation) as an indicative evaluation of imputation performance. The results of this can be seen in Supplementary Material, Table S1 for the AA data on subjects in the ‘African’ HLA-IMP-V2 reference data combined with our contributed AA samples. This table also contains *HLA-A, HLA-B* and *HLA-C;* however these analyses were performed on reference samples outside of our study.

We also performed our own imputation accuracy assessment on the 308 HLA-typed subjects that were also included in our association study. These results can be seen in Supplementary Material, Table S2–Supplementary Material, Table S4. This assessment is biased upwards for accuracy estimation, as the samples tested were also in the reference panel. However. the results are comparable with that returned by HLA^*^IMP-V2, which performed leave one-third out cross-validation on data that included our samples, with *HLA-DRB1* performing slightly worse than *HLA-DQA* and *HLA-DQB*.

### Amino acid translation

Amino acid sequences for each HLA allele were extracted from the European Bioinformatics Institute HLA database (http://www.ebi.ac.uk/ipd/imgt/hla/). HLA allele dosages were converted to amino acid dosages at each position; the dosage for a particular amino acid ‘A’ at position ‘p’ would be the sum of HLA alleles’ dosage that coded for amino acid ‘A’ at position ‘p’. The total dosage for each position is therefore equal to 2 and this total is split between each possible amino acid possible at the position.

### Phasing

The HLA data were phased together with the SNP data using BEAGLE (23) to aid the classical statistical analysis of the SLE HLA risk haplotypes.

### AA admixture analysis

The AA data were subject to an analysis for admixture using ADMIXTURE (24) on an LD-pruned dataset containing the AA samples as well as Hapmap3 (CEU, CHB and YRI) samples as anchoring populations. The resulting admixture estimates were used to remove genetic outliers. We also used this analysis to infer a set of subjects with a lower content of non-African derived haplotypes. This analysis was performed on genome-wide SNP data and on MHC-wide SNP data; results can be seen in Supplementary Material, Figure S4. The set of subjects chosen for HLA typing was all within the African cluster in the MHC-wide admixture analysis. We created a ‘more African’ subset of the AA data by removing AA subjects that were in the top 25th percentile of the non-African derived haplotypes estimate, which would have retained all Africans in the HapMap data; the data consisted of 1375 cases and 5414 controls. We refer to these data as **AA**_**sub**._

### Statistical analysis

#### Study design

**Figure 4:**
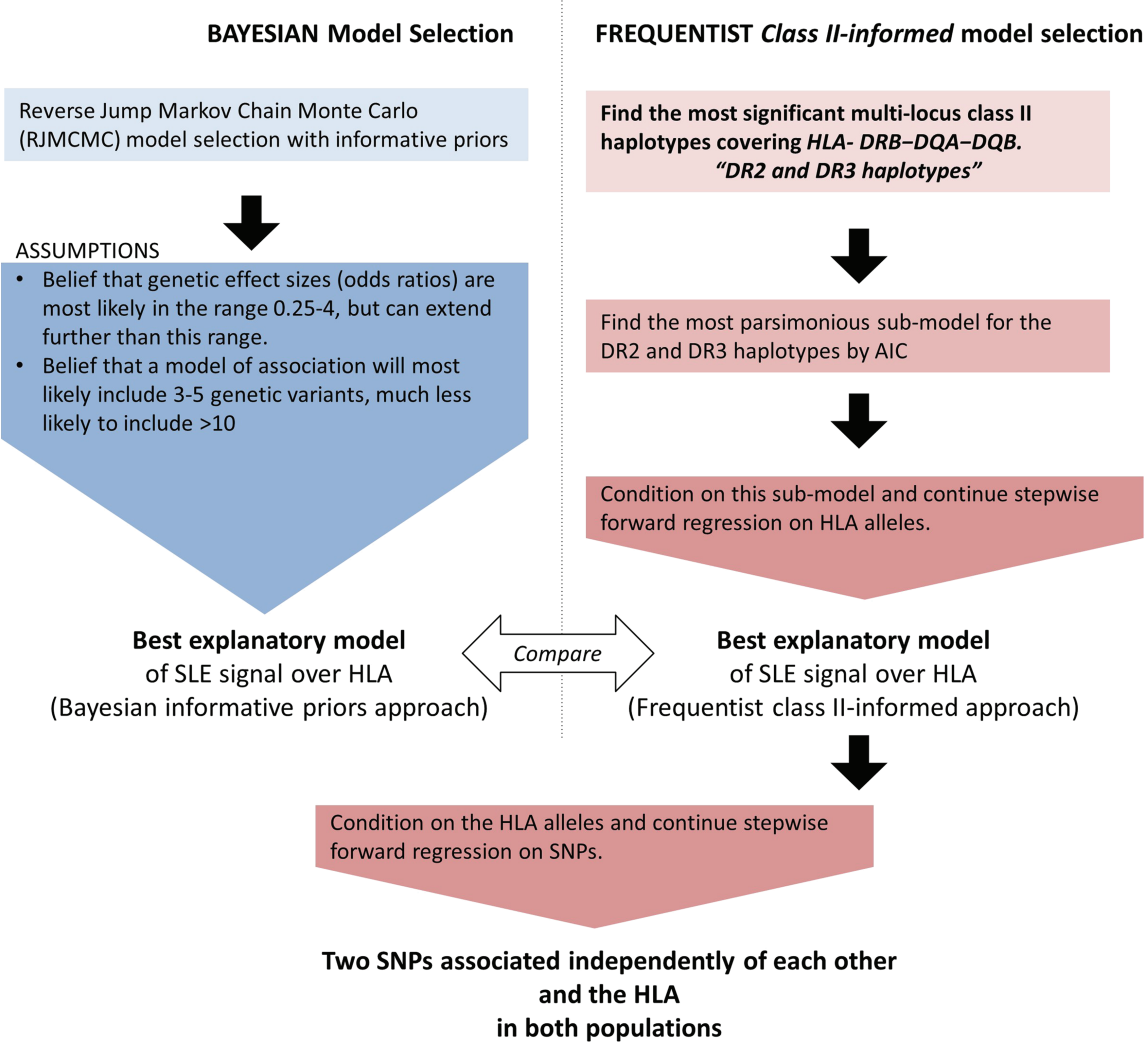
**Work flow chart for analyses strategy.** To determine the best model of association over HLA alleles, a Bayesian approach to model selection (left) was taken in parallel to a classical model choice (right). Following this a classical stepwise regression on SNPs was performed conditional on HLA alleles returned by the model choice.

We began with parallel frequentist and Bayesian association tests to determine the best underlying HLA risk model for SLE. After determining the best model of association at the HLA, we conditioned on this model, using classical stepwise regression, and tested for further association with SNPs. A workflow can be seen in Figure 4; we expand on each step in the description below. We also tested for association with SLE sub-phenotypes using classical stepwise regression.

#### Association analysis

Association analyses were performed in R (25) using logistic regression. SLE status was coded as 0 (Healthy controls) and 1 (cases). The SNP and HLA data were coded as minor allele counts (0 < g < 2) with imputed SNPs and HLA alleles coded as expected allele counts where the expectation was taken from the imputation probabilities: Expectation = 0 X P(G = 0) + 1 X P(G = 1) + 2 X P(G = 2), where P(G = j), for j = 0,1,2, is the probability of 0, 1 or 2 copies of the HLA or SNP reference allele. These probabilities were taken from the output of HLA^*^IMP V2. Covariates derived from an admixture analysis using ADMIXTURE (24) were used to account for population structure in the AA data. Our AA data were combined with HapMap European (CEU), African (YRI) and Asian (CHB + JPT) populations and we used the admixture proportions of CEU and YRI as covariates (the third proportion, assumed to be of Asian ancestry, being redundant as all sum to 1).

#### Analysis of extended MHC haplotypes

We used likelihood ratio testing between nested models of association with each of the SLE-associated class II haplotypes to find the best set of alleles that explained the association. This was complimented by checking the AIC and BIC for each model.

For example, in Supplementary Material, Table S6 where we look at the DRB^*^15:03—DQA^*^01:02—DQB^*^06:02 haplotype in the full AA data, we see that a simple model with a DQA^*^01:02—DQB^*^06:02 haplotype is rejected in favour of a model with the addition of DRB^*^15:03 as an extra explanatory variable (*P*-value = 5.92 × 10^−10^), likewise a simple model with a DRB^*^15:03—DQB^*^06:02 haplotype is rejected in favour of a model with the addition of DQA^*^01:02 as an extra explanatory variable (*P*-value = 2.04 × 10^−02^). So in both cases the addition of DRB^*^15:03 or DQA^*^01:02 is favoured. In the first case the addition of DRB^*^15:03 results in a lower AIC and BIC, while in the second case the addition of DQA^*^01:02 results in a lower AIC but not a lower BIC. However, at this four-digit resolution the best model as judged by the AIC is the model with DRB^*^15:03 + DQA^*^01:02 as separate additive explanatory variables, while the BIC is the lowest for inclusion of only DRB^*^15:03. At two-digit resolution for DRB^*^15; however the model with DRB^*^15: + DQA^*^01:02 as separate additive explanatory variables is best as judged by the BIC and AIC.

#### Stepwise regression.

 Forward stepwise regression was used to select markers as independently associated with SLE. We used a simple forward stepwise procedure and used an MHC-wide threshold as follows: for the AA data, at each stage of the stepwise search we used a significance threshold of *P*-value < 9.69 × 10^−06^, which controlled the MHC-wide testing type-one error rate at 0.01. The effective number of tests was estimated using the eigenvalue decomposition of the correlation matrix for the entire set of 4222 SNPs as in Li and Ji (2005) (26). This gave an estimate of 1032 effective tests resulting in a Bonferroni threshold of 9.69 × 10^−06^ if setting the family wise type-one error rate to be 0.01. For the EUR GWAS data, which had 6079 genotyped SNPs, the Bonferroni threshold was *P*-value < 1.15 × 10^−05^ (886 effective tests). It is not surprising that the AA data has a higher number of effective tests even though there are less genotypes markers as this population is well known to be more outbred and therefore having less LD across the genome (27).

#### Bayesian association analysis

 A Bayesian model selection was performed on the HLA data using the association studies toolkit for WinBUGS, employing a reverse jump algorithm on the model space, in the Markov Chain Monte Carlo (MCMC) framework (28). This approach used a probit link (rather than a logit link commonly used for case control association studies). The advantage is that the MCMC algorithm samples from an underlying normally distributed variable (z_i_) where the probability of disease for subject i is defined as p(z_i_ > 0 | M_i_) where the mean parameter M_i_ depends on a regression on the genotype values: M_i_ = beta ^*^ G_i,_ with G_i_ the genotype (the number of minor alleles for individual i) and beta is the regression parameter. We made simple prior assumptions: first that the magnitude of genetic effect (Odds ratio) could with non-negligible probability be in the range 0.25–4, and second that the genetic model would be most likely to have 3–5 genetic effects but much less likely to have more than 10 effects. We therefore used a Poisson distribution with mean parameter equal to 4; however we tested the robustness of our approach by re-running the analyses with Poisson (3) and Poisson (5). For the prior on the effect sizes we used a normal distribution with mean = 0 and variance = 0.25. This reflects the belief that the beta parameter is relatively unlikely to be larger than 1 (two standard deviations in our prior). A value of 1 on the probit scale, with samples sizes similar to the ones in our study, transfers to a relative risk of ∼1.7 and so most of our prior belief in the relative risk is between 0.5–2, while values below 0.5 and above 2 are allowed but with less belief. It is important to have informative priors in Bayesian model choice as vague priors can overly favour the null model (zero effect size or equivalently no explanatory variables in the chosen model). Our priors are informative but not overly so, reflecting the commonly observed risk effects in GWAS.

The MCMC model fitting in WinBUGS is a computationally expensive exercise; however it was feasible within a period of 2 days to get results. The MCMC framework is a sampling-based technique that requires convergence. With the current AA and EUR data we found that running six chains in parallel each of 80 000 samples with a burn-in period (where samples are discarded) of 20 000 was sufficient. This required a 12-core desktop PC with two 2.4 GHz Xeon processors and utilized 10GB of RAM.

#### The HLA-DQ heterodimer risk profile

 We tested for interaction between all *DQA-DQB* pairs noted in Figure 2. For example, in the case of EUR we tested for interaction between DQA^*^01:02 and DQB^*^02:01.

We also created a variable from the product of the two DQA and DQB pairs and tested this as a sole variable in the regression; we then compared the AIC and BIC for this single-variable model to the two-parameter models (independent additive effect for the DQA and DQB alleles). This single-parameter model captures risk attributable to the specific DQ molecules created by the pairing, for example, the variable created from the product of DQA^*^01:02 × DQB^*^02:01 gives the expected number of DQA^*^01:02/DQB^*^02:01 molecules that could be expressed by an individual: an individual with one copy of DQA^*^01:02 and two copies of DQB^*^02:01 can make two molecules consisting of DQA^*^01:02/DQB^*^02:01, while an individual with two copies of DQA^*^01:02 and two copies of DQB^*^02:01 can make four molecules consisting of DQA^*^01:02/DQB^*^02:01.

#### Testing for interaction.

 We tested for interaction between the associations for the two HLA alleles (DQA*02:01 and DQB*02:02) by adding an interaction term in the multiple logistic regression model with the two alleles as explanatory variables.

#### Sub-phenotype analysis.

We performed conditional association analyses (forward selection) on each sub-phenotype, in AA and EUR. These analyses were case-only (presence or absence of the antibody in cases, healthy controls not used) on genotyped SNPs and HLA alleles combined. Anti-Ro, anti-La, anti-Sm and anti-RNP autoantibody sub-phenotypes were available in both the AA (N = 1200) and EUR (N = 2310) data.

### Ethics

Ethical approval was obtained from the institutional review committee of King’s College London (Study Ref: 07/H0718/049). All patients with SLE and healthy controls were given information sheets and verbal explanations of what the research entailed. Informed written consent was obtained from all subjects.

## Web resources

Summary association data for this study are available at http://insidegen.com/insidegen-LUPUS-data.html

## Acknowledgements

We would like to thank the original study participants and their families for their contributions to this research, along with clinical colleagues who facilitated data collection.

*Conflict of Interest statement*. None declared.

## Funding

NIH (R01 AI024717, U01 HG008666, P30 AR070549, P01 AI08394 and R01 AR042460 to J.B.H., P01 AR49084 and R01 AR064820 to R.P.K. and E.E.B., P01 AR049084, K24 AR 00218, P60AR 064464 (formerly P60 AR30692), UL1TR001422 (formerly ULRR025741) to R.R.-G., R01 AR056360, AR063124, P30 GM110766 and U19 AI082714 to P.M.G., U19AI082714, U01AI101934, P30GM103510, U54GM104938, P30AR053483 to J.A.J.); the US Department of Veterans Affairs (I01 BX001834 to J.B.H.); US Department of Defense (PR094002 to J.B.H). National Institute for Health Research Biomedical Research Centre (NIHR BRC) at Guy’s and St Thomas’ NHS Foundation and King’s College London and by the NIHR BRC at South London and Maudsley NHS Foundation Trust and King’s College London.

## Author contributions

J.A.N. performed the HLA genotyping in the AA subjects. D.L.M. and P.T. performed the quality control on the EUR and AA genotype data. D.L.M. performed SNP imputation on the AA and EUR data. C.D.L., K.M.K. and M.C. performed the admixture analysis in the AA data. T.J.V., C.M.L. and D.L.M. supervised the study and analysis plan. D.L.M. and A.T.D. performed HLA imputation on the AA and EUR data. D.L.M. phased the AA and EUR SNP-HLA alleles. K.H. and D.L.M. performed the haplotype analysis. K.H. and D.L.M. performed the classical stepwise regression. D.L.M. performed the Bayesian RJMCMC analysis. K.H. and D.L.M. performed the sub-phenotype analysis. K.H., D.L.M., J.A.N., K.L. and T.J.V. wrote the manuscript. K.M.K., M.E.A.-R., P.M.G., C.O.J., K.M.-S., B.P.T., G.A., E.B., J.C., J.E., G.G., J.A.J., D.K., J.A.K., J.M.C., J.M., M.P., R.R.-G., J.D.R., J.S., H.S., T.U., D.W., M.W., R.P.K., J.B.H., L.A.C. and T.J.V. contributed to data acquisition through clinical recruitment, clinical characterization, sample processing and autoantibody testing.

## Supplementary Material

Supplementary Data
