# Supplementary material for: Genetic fine mapping of systemic lupus erythematosus MHC associations in Europeans and African Americans
Source: Hum Mol Genet. 2018 Jul 31;27(21):3813–24. doi: 10.1093/hmg/ddy280 (PMC6196648; doi:10.1093/hmg/ddy280)
Supplement: Supplementary Data [file ddy280_supp.zip › AA_EUR_SLE_MHC_Association_16072018 supp.docx]

**Genetic fine mapping of Systemic Lupus Erythematosus MHC associations in Europeans and African Americans**

**Supplementary Material**

**Ken B Hanscombe^1,2^, David L Morris^1,2^, Janelle A Noble^3^, Alexander T Dilthey^4,*^, Philip Tombleson^2^, Kenneth M Kaufman^5^, Mary Comeau^6^, Carl D Langefeld^6^, Marta E Alarcon-Riquelme^7,8^, Patrick M Gaffney^9^, Chaim O Jacob^10^, Kathy L Sivils^9^, Betty P Tsao^11^, Graciela S Alarcon^12^, Elizabeth E Brown^13^, Jennifer Croker^14^, Jeff Edberg^12^, Gary Gilkeson^15^, Judith A James^9,24^, Diane L Kamen^15^, Jennifer A. Kelly^9^, Joseph McCune^16^, Joan T Merrill^17^, Michelle Petri^18^, Rosalind Ramsey-Goldman^19^, John D. Reveille^20^, Jane E Salmon^21^, Hal Scofield^9,22^, Tammy Utset^23^, Daniel J Wallace^24^, Michael H Weisman^24^, Robert P Kimberly^12^, John B Harley^5^, Cathryn M Lewis^2,25^, Lindsey A Criswell^26^ and Timothy J Vyse^2^**

^1^ These authors contributed equally to this work

^2^ Department of Medical and Molecular Genetics, King’s College London, London, UK

^3^ CHORI, Children’s Hospital Oakland Research Institute, Oakland, California, USA

^4^ Wellcome Trust Centre for Human Genetics, University of Oxford, UK

^5^ Center for Autoimmune Genomics and Etiology (CAGE), Department of Pediatrics, Cincinnati Children’s Medical Center & University of Cincinnati and the US Department of Veterans Affairs Medical Center, Cincinnati, Ohio 45229, USA.

^6^ Center for Public Health Genomics, Wake Forest School of Medicine, Winston-Salem, North Carolina, USA^.^

^7^ Pfizer-University of Granada-Junta de Andalucía Centre for Genomics and Oncological Research (GENYO), Granada, Spain

^8^ Unit of Chronic Inflammation, Institute of Environmental Medicine, Karolinska Institute, Sweden.

^9^ Arthritis & Clinical Immunology Research Program, Division of Genomics and Data Sciences, Oklahoma Medical Research Foundation, Oklahoma City, Oklahoma, USA

^10^ Keck School of Medicine of USC, Los Angeles, California, USA.

^11^ Department of Medicine, Medical University of South Carolina, Charleston, South Carolina, USA

^12^ Division of Clinical Immunology and Rheumatology, University of Alabama at Birmingham, Birmingham, Alabama, USA

^13^ Department of Pathology, University of Alabama at Birmingham, Birmingham, Alabama, USA

^14^ Center for Clinical and Translational Science, University of Alabama at Birmingham, Birmingham, Alabama, USA

^15^ Division of Rheumatology, Medical University of South Carolina, Charleston, USA.

^16^ Michigan Medicine Rheumatology Clinic, Taubman Center Floor 3 Reception A, 1500 E Medical Center Dr SPC 5358, Ann Arbor, MI 48109-5358

^17^ Oklahoma Medical Research Foundation, 825 N.E. 13th Street, Oklahoma City, OK 73104

^18^  Division of Rheumatology, Department of Medicine, Johns Hopkins Medicine

^19^ Feinberg School of Medicine, McGaw Pavilion Suite M-300, 240 E Huron, Chicago IL 60611

^20^ **The University of Texas. Department of Internal Medicine, 6431 Fannin, MSB 1.150, Houston, Texas 77030,**

^21^ Division of Rheumatology, Hospital for Special Surgery-Weill Cornell Medicine, New York, NY

^22^ Oklahoma Clinical and Translational Science Institute, University of Oklahoma Health Sciences Center, 920 NE Stanton L. Young, Oklahoma City, OK 73104, USA

^23^ University of Chicago Pritzker School of Medicine, Chicago, Illinois, USA.

^24^ Division of Rheumatology, Cedars Sinai Medical Center, Los Angeles, USA

^25^ MRC Social, Genetic and Developmental Psychiatry Centre, Institute of Psychiatry, Psychology & Neuroscience, King’s College London, London, UK

^26^ Rosalind Russell / Ephraim P Engleman Rheumatology Research Center, Division of Rheumatology, UCSF School of Medicine, San Francisco, California, USA

*** Current address:** National Human Genome Research Institute, National Institutes of Health, 9000 Rockville Pike, Bethesda, Maryland 20892-2152, USA

**Contents**

**Supplementary Notes Pages 4-6**

**Figure S1 Page 7**

**Figure S2 Page 8**

**Figure S3 Page 9**

**Figure S4 Page 10 - 15**

**Figure S5 Page 16**

**Table S1 Page 17**

**Table S2 Page 18**

**Table S3 Pages 19-20**

**Table S4 Pages 21-22**

**Table S5 Pages 23-24**

**Table S6 Page 25-26**

**Table S7 Page 27**

**Table S8 Pages 28-29**

**Table S9 Page 30-31**

**Table S10 Pages 32-33**

**Supplementary Notes**

**1. Analysis of extended MHC haplotypes** The limited LD in the AA data around the association peak in the class II region motivated a stepwise approach beginning at this locus. A model of association at class II in both populations had first to be determined however, before conditioning on this to find further independent association across the MHC. Our analysis (covered in methods) used likelihood ratio testing together with the AIC and BIC model choice criteria to determine the best model of association considering all alleles and sub-haplotype pairs within each of the class II haplotypes discussed below.

The most significant multi-locus class II haplotype in AA_sub_ was *DRB*15:03*—*DQA*01:02*—*DQB*06:02* (p = 7.18x10^-22^, OR = 1.74, see **Supplementary Table 5**), and the most parsimonious model to account for this class II signal in AA_sub_ included only the two alleles *DRB*15:03* and *DQA*01:02*. The most parsimonious account of the class II signal in full AA data (**Supplementary Table 6)** was also the two alleles *DRB*15:03* and *DQA*01:02.* The corresponding multi-locus haplotype in EUR (*DRB*15:01* replacing *DRB1*15:03*) was also significant (p = 8.23x10^-10^, OR = 1.30) (**Supplementary Table 7)**. We found that the most parsimonious account of this class II signal in EUR included only the two alleles *DRB*15:01* and *DQA*01:02,* as in the AA cohort*.*

Although *DRB*15:01* is strongly associated in EUR, the most significant multi-locus class II haplotype in EUR was *DRB*03:01*—*DQA*05:01*—*DQB*02:01* (p = 2.58x10^-95^, OR = 2.32, see **Supplementary Table 8**). The most parsimonious model to account for this class II signal in EUR was the single allele *DQB*02:01*. This multi-locus haplotype did not pass multiple testing correction in AA_sub_ (p = 3.42x10^-03^, OR = 1.27), however we found that the most parsimonious account of this class II signal in full AA data (**Supplementary Table 9)** and the AA_sub_ data (**Supplementary Table 10)** was the single allele *DQA*05:01.* However, in both populations the most parsimonious two-allele model was that comprising the combination of both HLA-DQ alleles: *DQA*05:01- DQB*02:01.*

We therefore find that the best model for association at the class II loci are as follows:

AA: *DQA*05:01 + DRB*15:03* + *DQA*01:02 (1)*

EUR: *DQB*02:01 + DRB*15:01* + *DQA*01:02 (2)*

**2. Conditional analyses – additional HLA alleles**  A stepwise regression using the European data with the alleles noted in **(2)** above as covariates identified three further alleles as statistically significant: *HLA-B*08:01, HLA-B*18:01* and *HLA-DR3*02:--.* A stepwise regression using the full AA data with the alleles noted in **(1) above** as covariates identified six further alleles as statistically significant: *HLA-B*08:01, HLA-C*17:01, HLA-DQA*02:01, HLA-DQA*05:05, HLA-DQB*02:02* and *HLA-DQB*03:19.* Except for *HLA-C*17:01* the same HLA alleles were significant in the more African subset (AA_sub_). This leads to the models of association displayed in **Figure 2b-iii**. Association results for the alleles in these models can be seen in **Table 1.**

**3. Bayesian model choice on HLA alleles**

We applied Reversible-Jump Markov Chain Monte Carlo (RJMCMC) simulation to explore the model space spanned by all the HLA alleles in the AA (301 observed alleles) and EUR (199 observed alleles) data using WinBugs (see methods). The best model fit to the EUR data consisted of the five HLA alleles *B*08:01 + B*18:01 + DQA*01:02 + DQB*02:01 + DRB3*02* and the best model in the AA data consisted the eight alleles *C*17:01 + B*08:01 + DRB1*15:03 + DQA*02:01 + DQA*05:01 + DQA*05:05 + DQB*03:19 + DQB*02:02* displayed in **Figure 2**. The posterior probabilities of being included in a model of association for each of the HLA alleles are displayed in **Figure S2 for** AA and EUR. The points in these figures represent the probability that an allele is associated, averaged over all possible models of association. The only alleles with probabilities greater than 0.5 are the ones listed above contained in the best models of association in each population. So the Bayesian model choice results point directly to these sets of alleles as together forming the best models explaining association in the HLA and marginally as the only alleles that we believe are independently associated.

The results for a “more African" subset of the AA data (AA_sub_, obtained by removing AA subjects that were in the top 25th percentile of the non-African derived haplotypes estimate, see methods) agreed with the full AA data (**Figure S2**) with the only noticeable difference being a near zero probability for inclusion of *HLA-DRB1*15:01* due to removal of European derived haplotypes. Therefore there was no difference between the results except for that due to the low frequency of *HLA-DRB1*15:01* in the African population.

We note that a simple forward stepwise regression on HLA alleles starting from just the covariates used to account for population structure (Null model, no HLA alleles), returned the same alleles as in the Bayesian analyses in the EUR data, while in the AA data only *DRB*15:03*, *DQA*05:01* and *B*08:01* were returned using the strict MHC-wide threshold (see methods). Relaxing the threshold to HLA allele wide (p < 3 x 10^-05^, Bonferroni on 301 tests with a test size of 0.01) also included *DQB*03:19* and *C*17:01*. The difference between this simple stepwise regression and the Bayesian approach therefore was the omission of *DQA*02:01, DQA*05:05* and *DQB*02:02* in the AA data.

**4. Class I HLA associations** *HLA-DRB1*03:01—HLA-DQA*05:01—HLA-DQB*02:01* is part of a longer multi-locus haplotype that extends to class I to include *HLA-A*01:01*, *HLA-Cw*07:01*, and *HLA-B*08:01*. The risk contributed by ancestral MHC 8.1 (AH8.1: also known as super B8, *HLA A1-B8-DR3-DQ2*) is typically associated with the *HLA-B*08:01* allele. We found that in all cases the addition of the class I allele *HLA-B*08:01* (AA p = 3.3 x 10^-07^, EUR p = 1.5x10^-16^) improved the model fit of the class II models referred to above, with a reduction of BIC (AIC) in the EUR data from 13614 (13571) to 13556 (13505), a reduction in BIC (AIC) from 7431 (7403) to 7411 (7377) in the AA data, and a reduction in BIC (AIC) from 6829 (6802) to 6828 (6783) in the AA_sub_ data, as presented in **Supplementary Tables 8-10**. The independent association of the class I allele is evidence from the association plot in **Figure 3 A and D** in the EUR and AA data, respectively.

We therefore find that the best model for association at the class II extending over the ancestral MHC 8.1 haplotype is

AA: *DQA*05:01 + DRB*15:03* + *DQA*01:02 + HLA-B*08:01 (3)*

EUR: *DQB*02:01 + DRB*15:01* + *DQA*01:02 + HLA-B*08:01 (4)*

We note that the same alleles in **Figure 2b-iii** are identified from a stepwise regression starting from the class II alleles noted in (3) for AA and (4) for EUR.

**
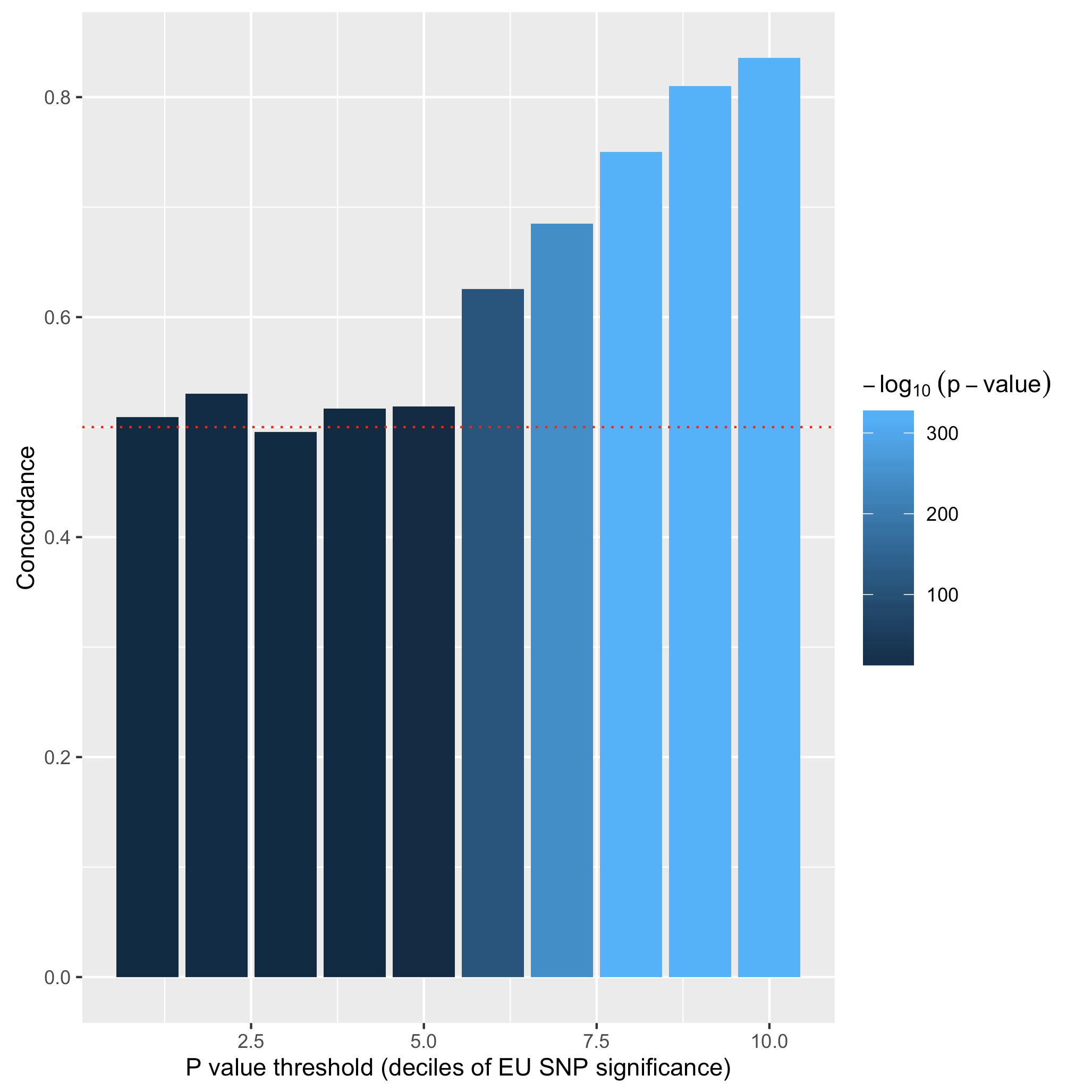

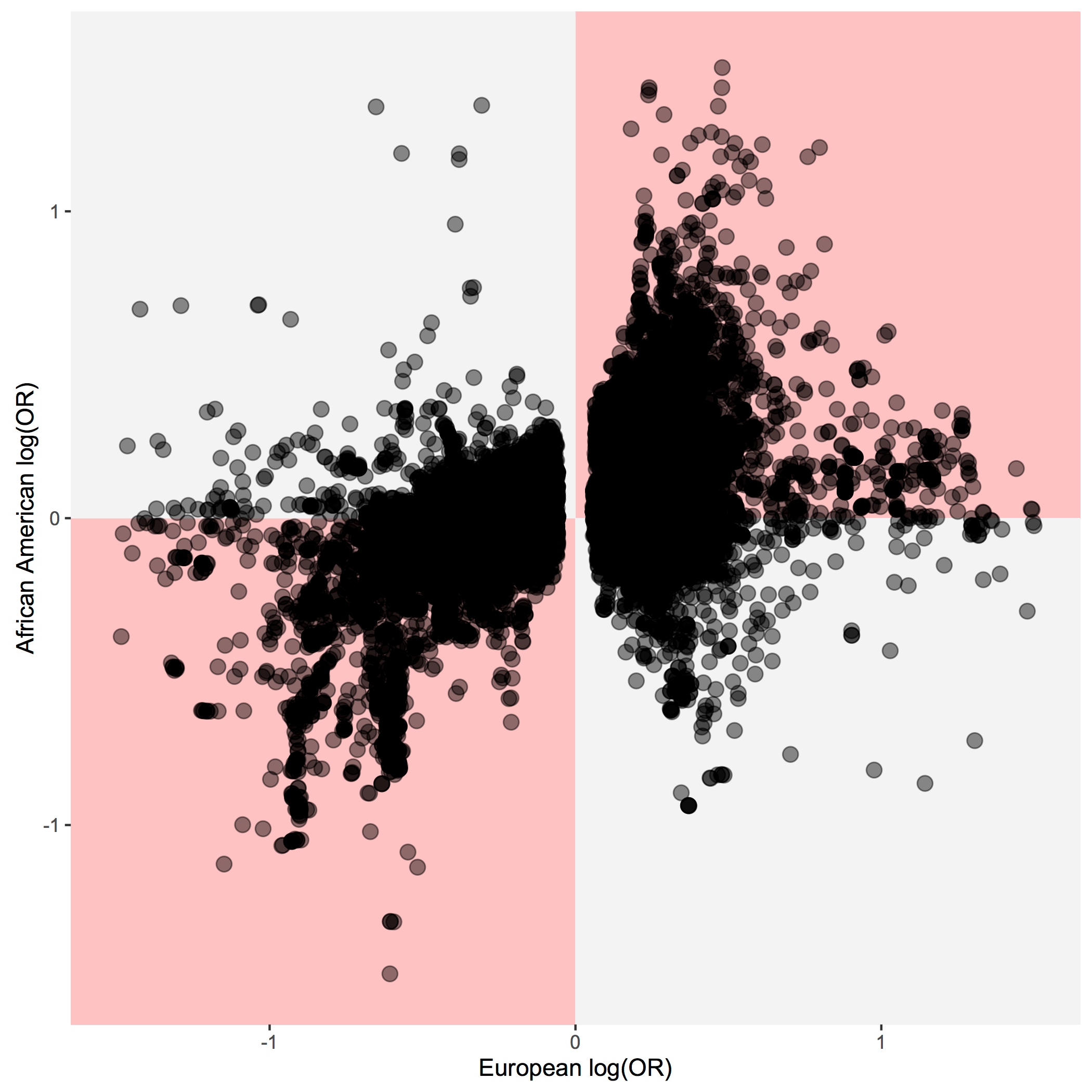
**

**Figure S1. Comparison of SNP associations between the AA and EUR data. (A) Concordance of log odds ratios at difference levels of statistical significance. At low levels of significance there is no evident agreement, while the more significant SNPs have high concordance. (B) A scatter plot of log odds ratios between AA and EUR.**

**
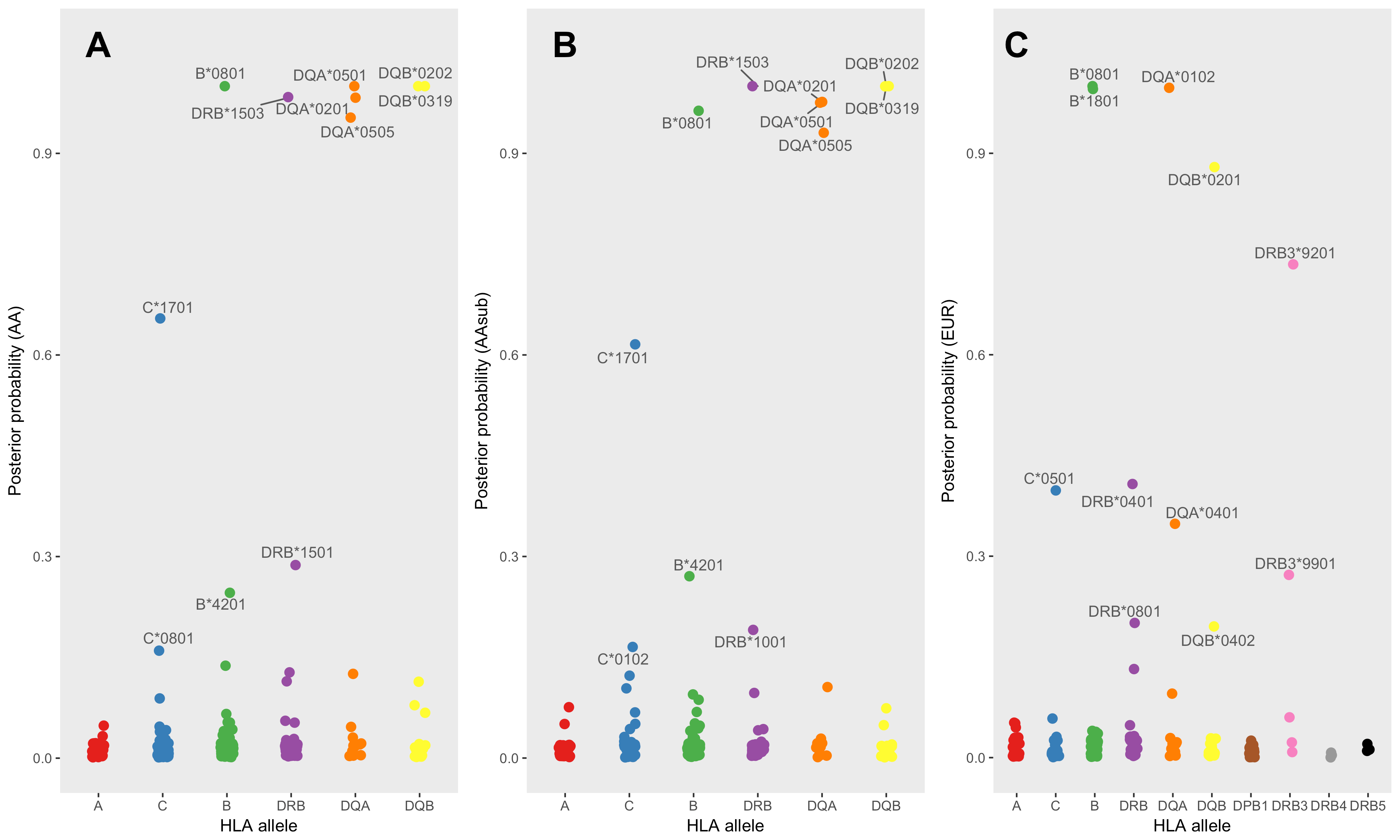
**

**Figure S2. Posterior probabilities of model inclusion for each HLA allele in the (A) African American (AA) data, (B) More African subset of the AA data, (C) European data. Each point represents the probability of the allele being included in any model of association.**

**
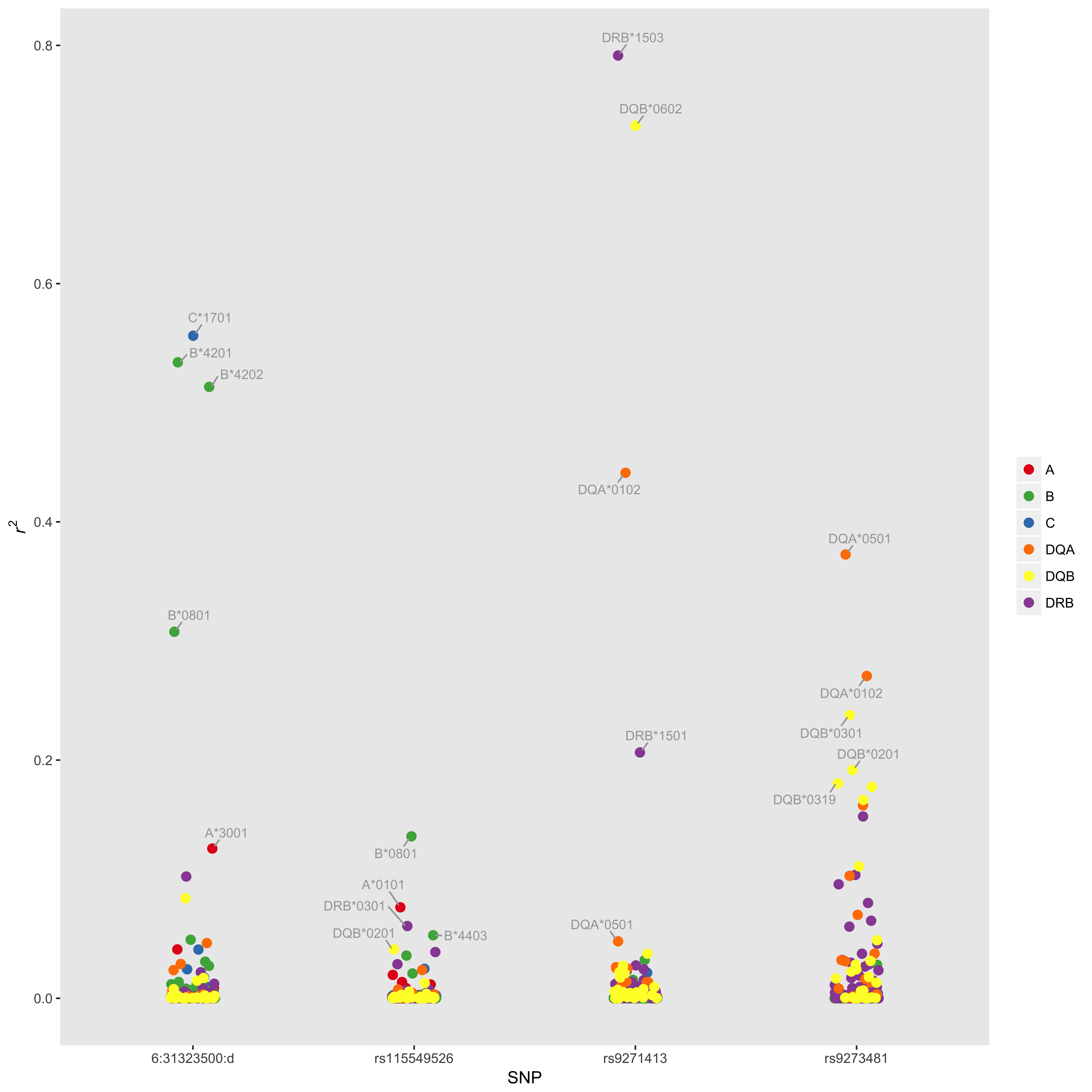

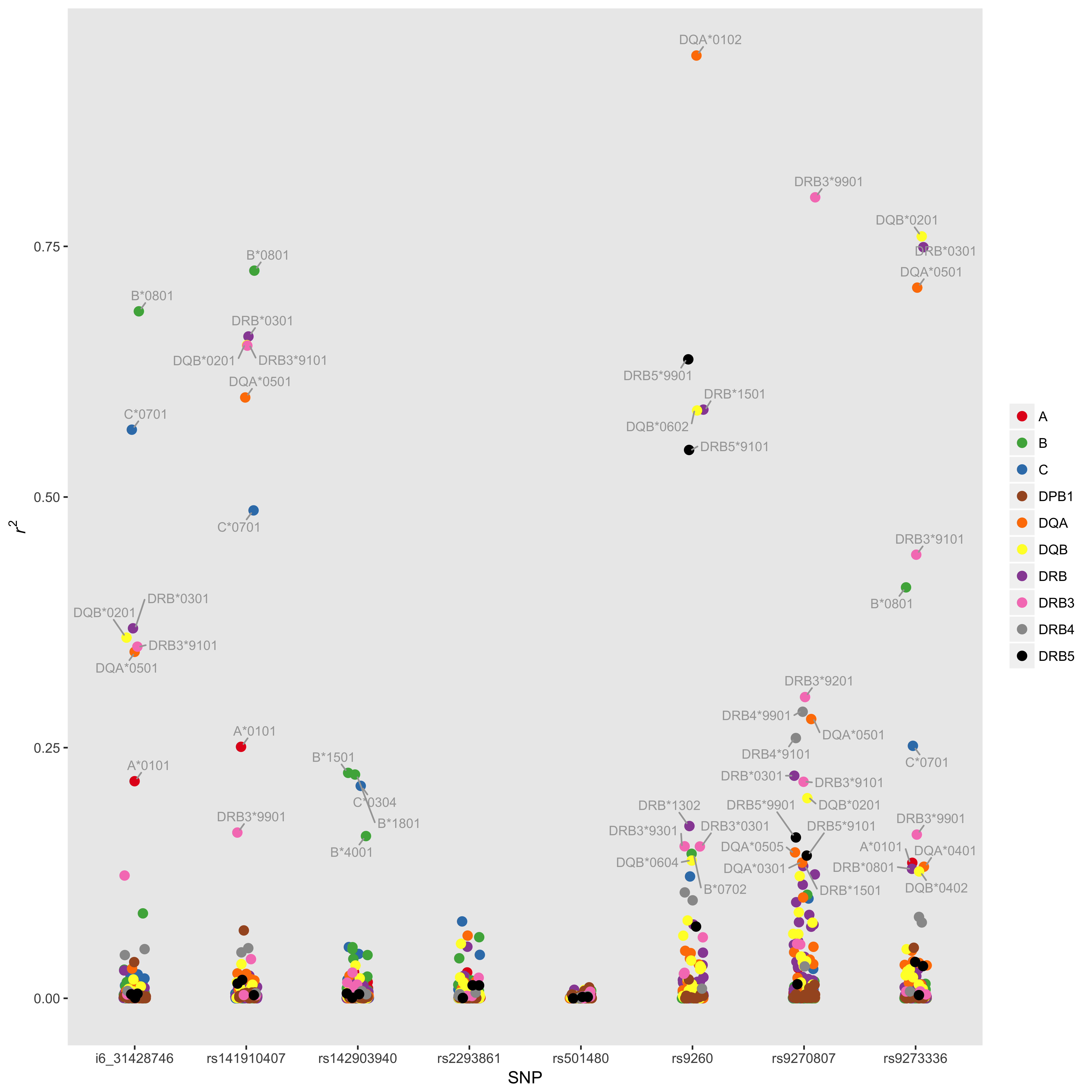
**

1. **(B)**

**Figure S3: HLA alleles tagged by SNPs returned in the SNP-only stepwise regression. (A) African American data. (B) European data**

**
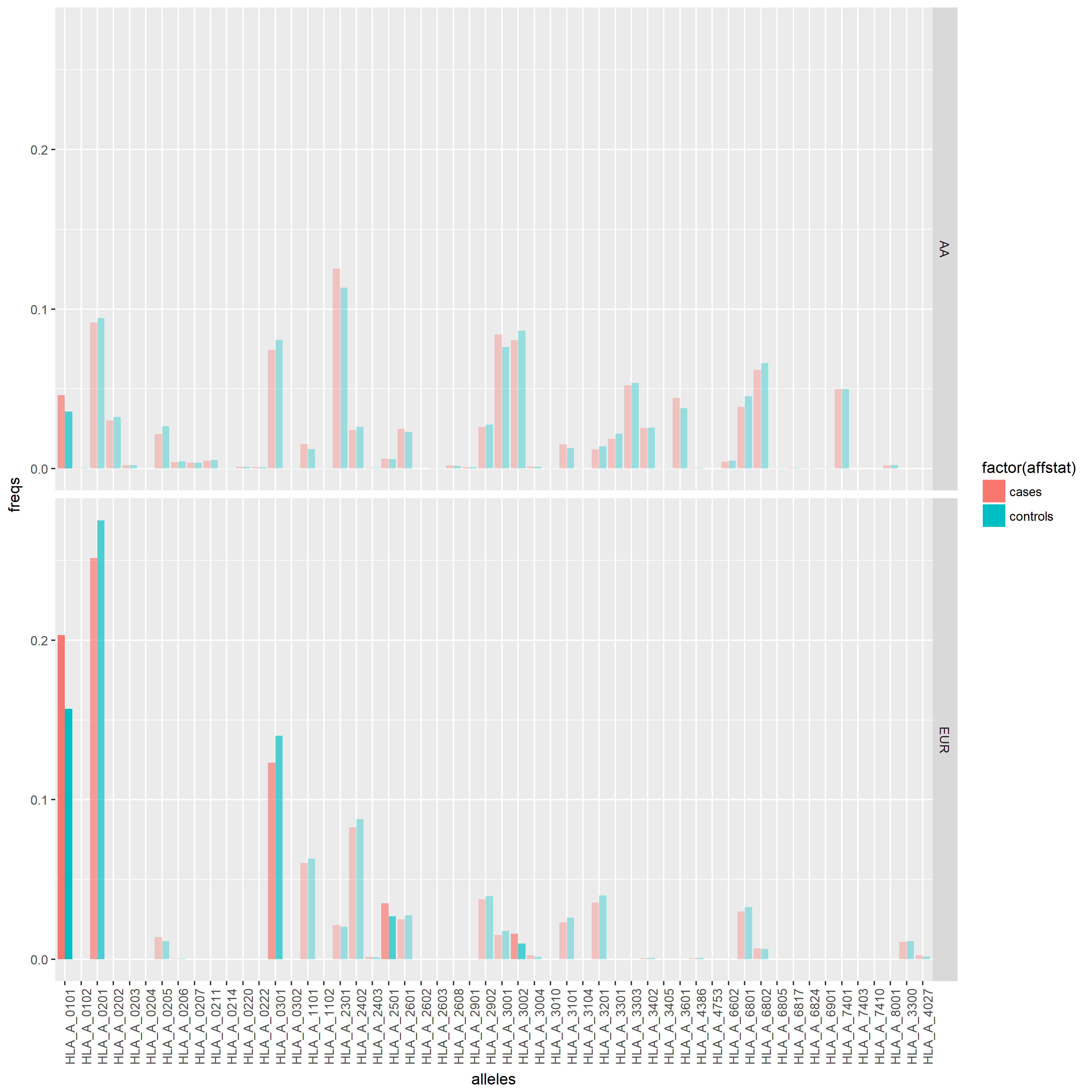
**

**Figure S4a. Association results for *HLA*A* alleles in the AA and EUR data. Allele frequencies in cases and controls are on the y-axis. Statistical significance is highlighted by boldness of fill colour. There are three levels of significance highlighted: very bold fill p < 1E-05; medium bold fill p < 0.01; weak fill: p > 0.01.**

**
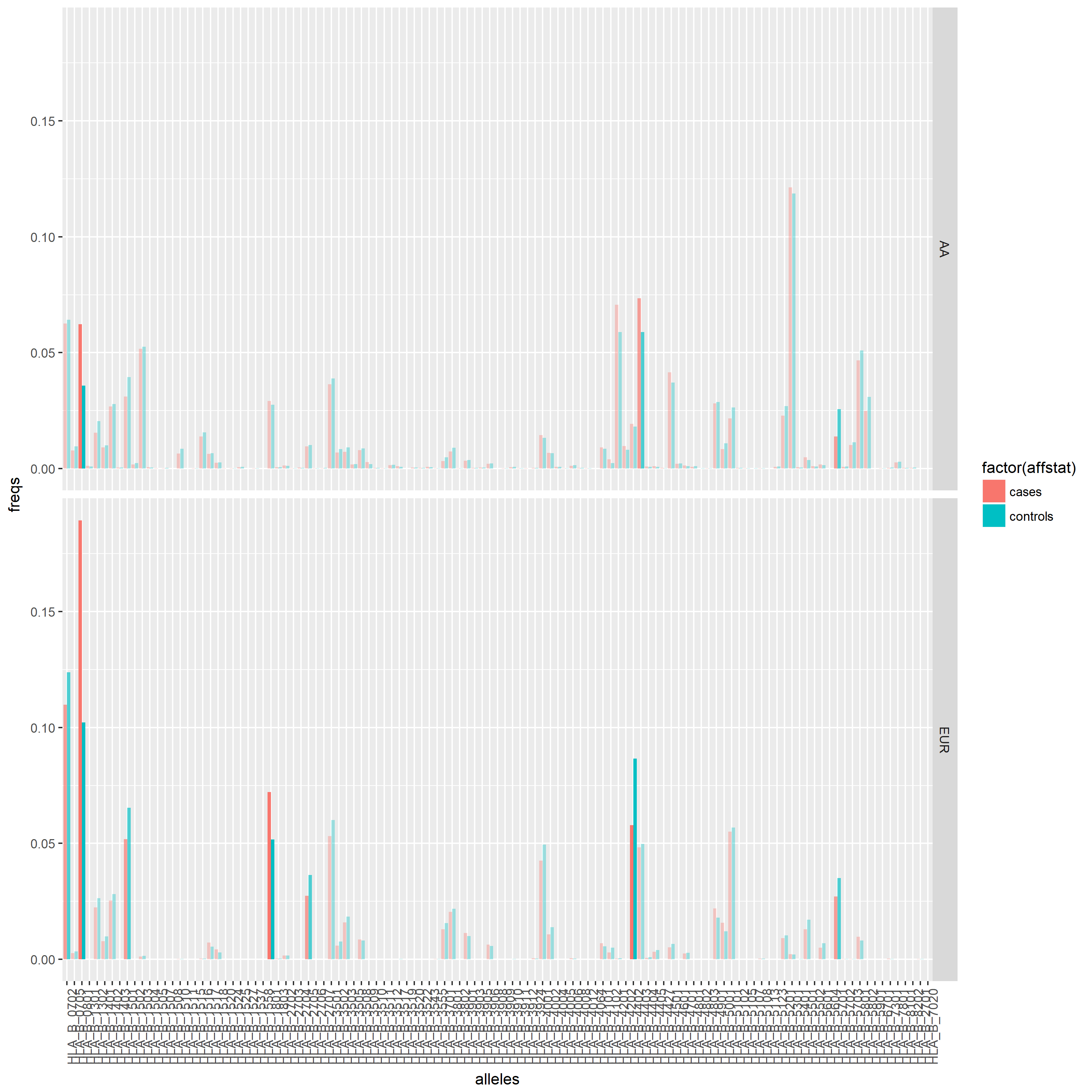
**

**Figure S4b. Association results for *HLA*B* alleles in the AA and EUR data. Allele frequencies in cases and controls are on the y-axis. Statistical significance is highlighted by boldness of fill colour. There are three levels of significance highlighted: very bold fill p < 1E-05; medium bold fill p < 0.01; weak fill: p > 0.01.**

**
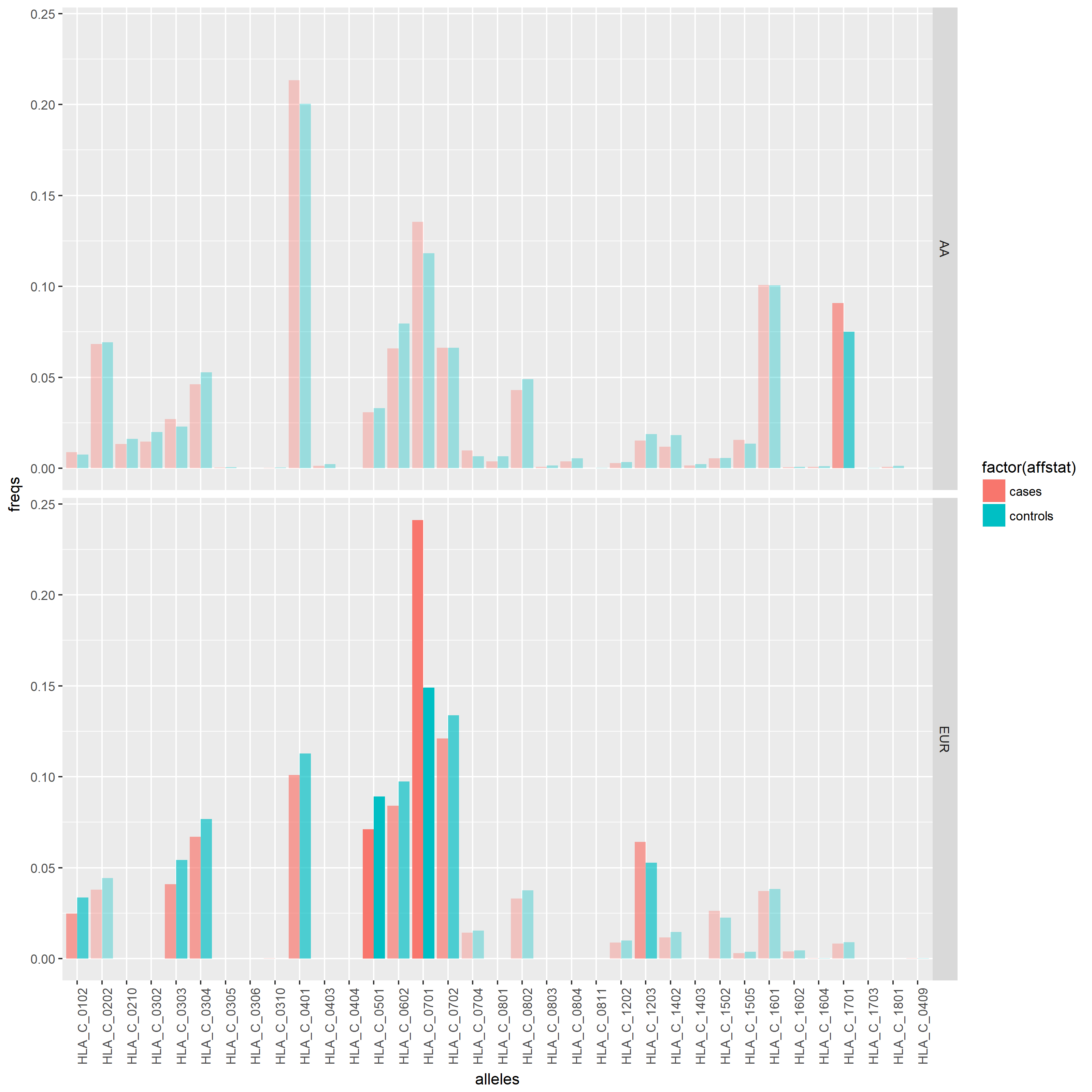
**

**Figure S4c. Association results for *HLA*C* alleles in the AA and EUR data. Allele frequencies in cases and controls are on the y-axis. Statistical significance is highlighted by boldness of fill colour. There are three levels of significance highlighted: very bold fill p < 1E-05; medium bold fill p < 0.01; weak fill: p > 0.01.**

**
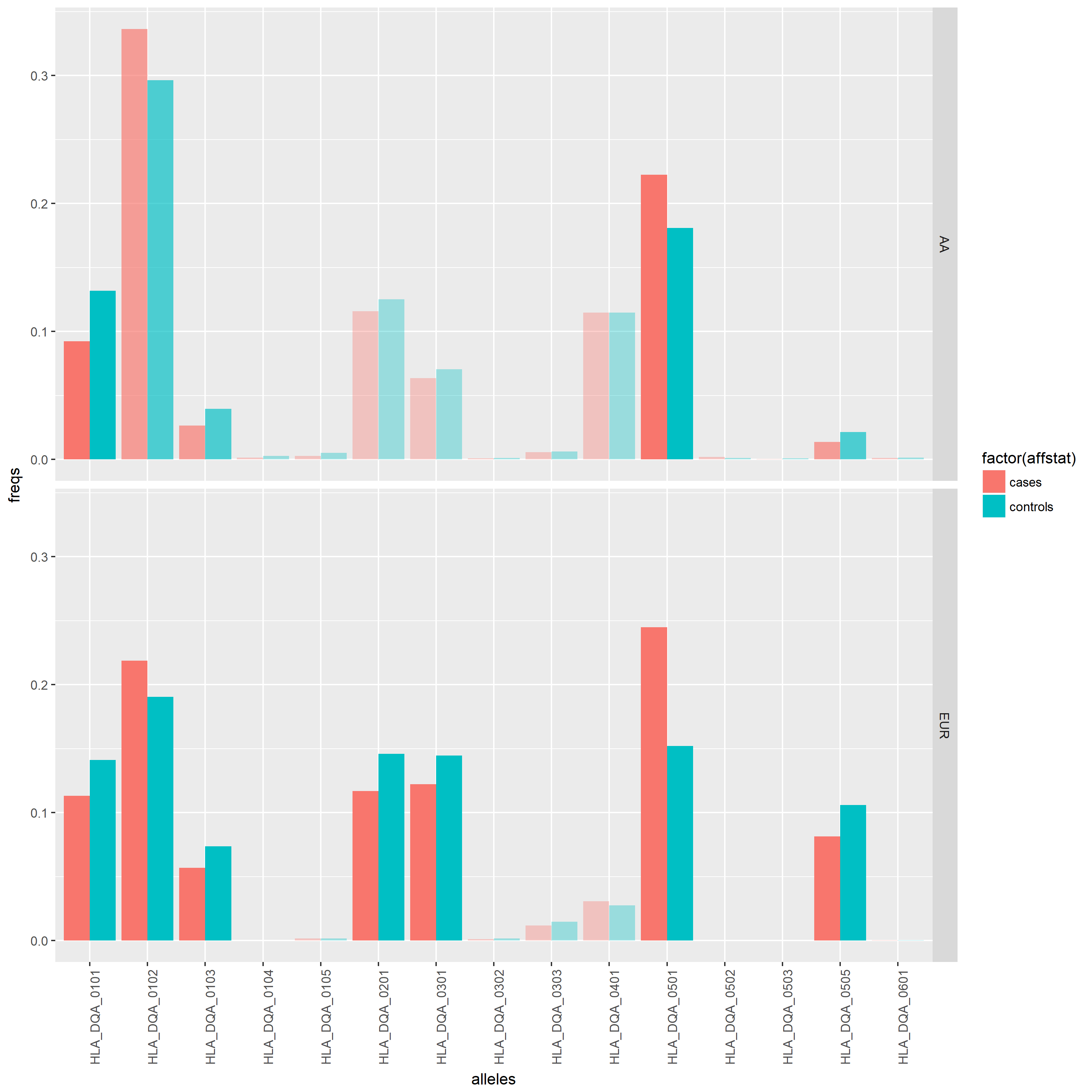
**

**Figure S4d. Association results for *HLA*DQA* alleles in the AA and EUR data. Allele frequencies in cases and controls are on the y-axis. Statistical significance is highlighted by boldness of fill colour. There are three levels of significance highlighted: very bold fill p < 1E-05; medium bold fill p < 0.01; weak fill: p > 0.01.**

**
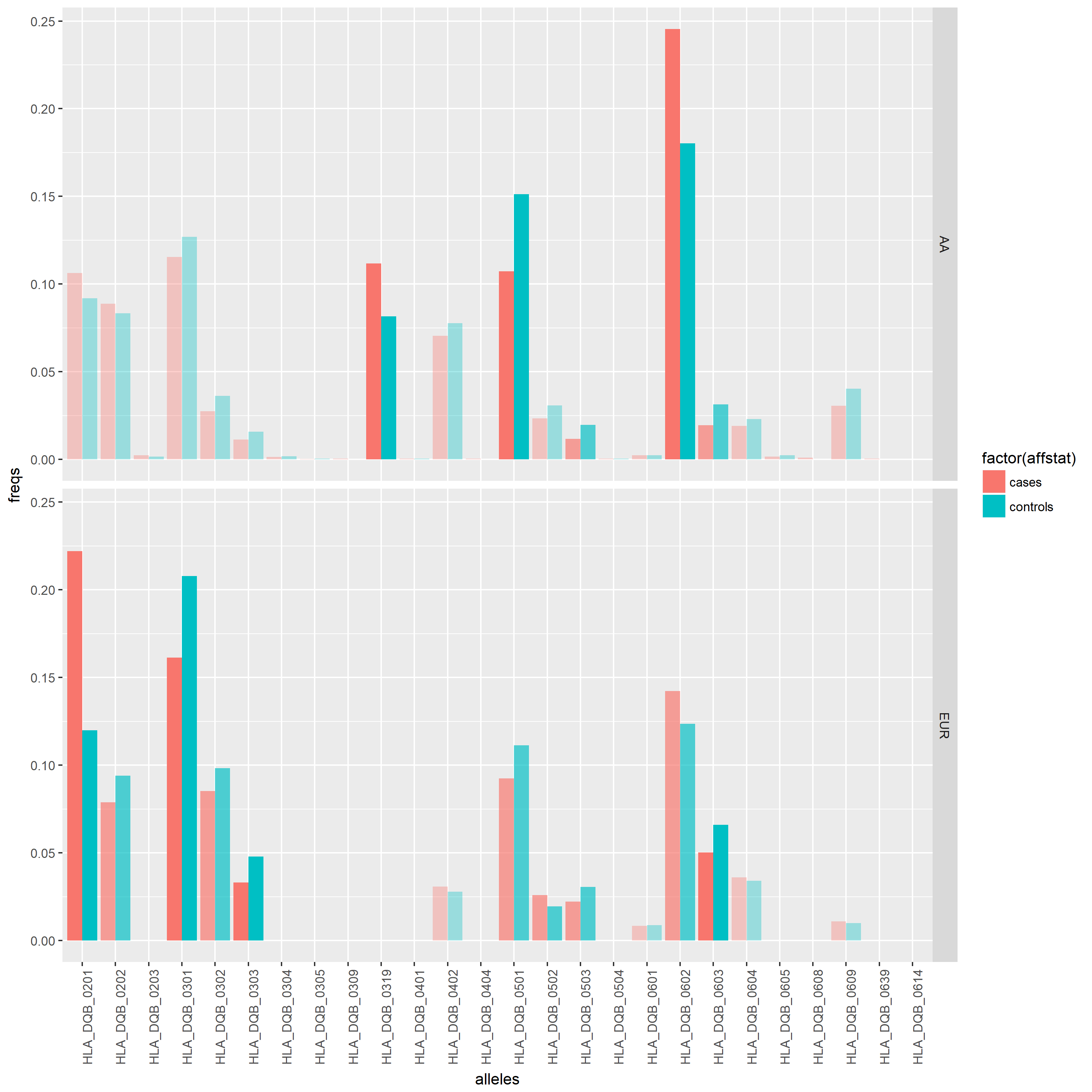
**

**Figure S4e. Association results for *HLA*DQB* alleles in the AA and EUR data. Allele frequencies in cases and controls are on the y-axis. Statistical significance is highlighted by boldness of fill colour. There are three levels of significance highlighted: very bold fill p < 1E-05; medium bold fill p < 0.01; weak fill: p > 0.01.**

**
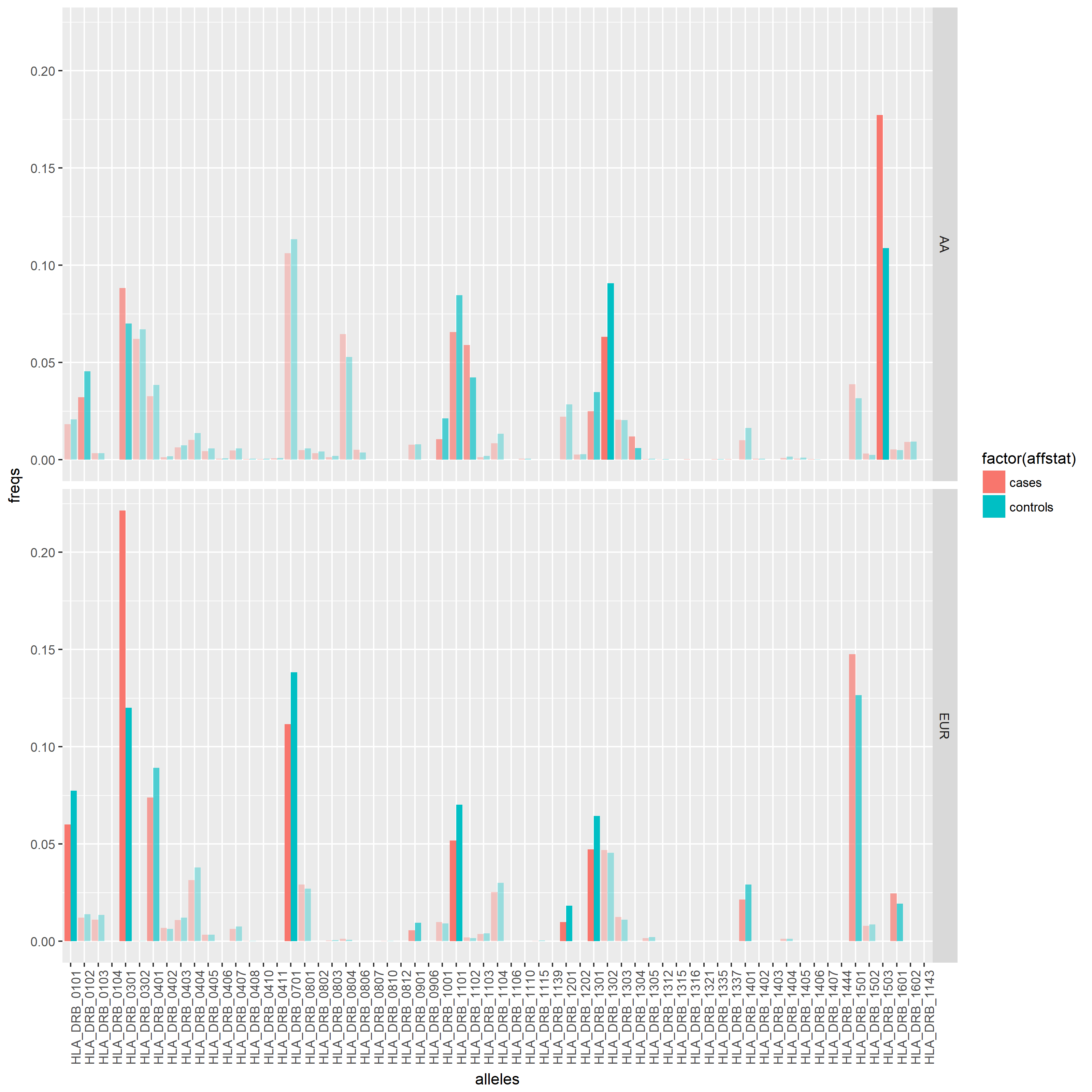
**

**Figure S4f. Association results for *HLA*DRB1* alleles in the AA and EUR data. Allele frequencies in cases and controls are on the y-axis. Statistical significance is highlighted by boldness of fill colour. There are three levels of significance highlighted: very bold fill p < 1E-05; medium bold fill p < 0.01; weak fill: p > 0.01.**

**
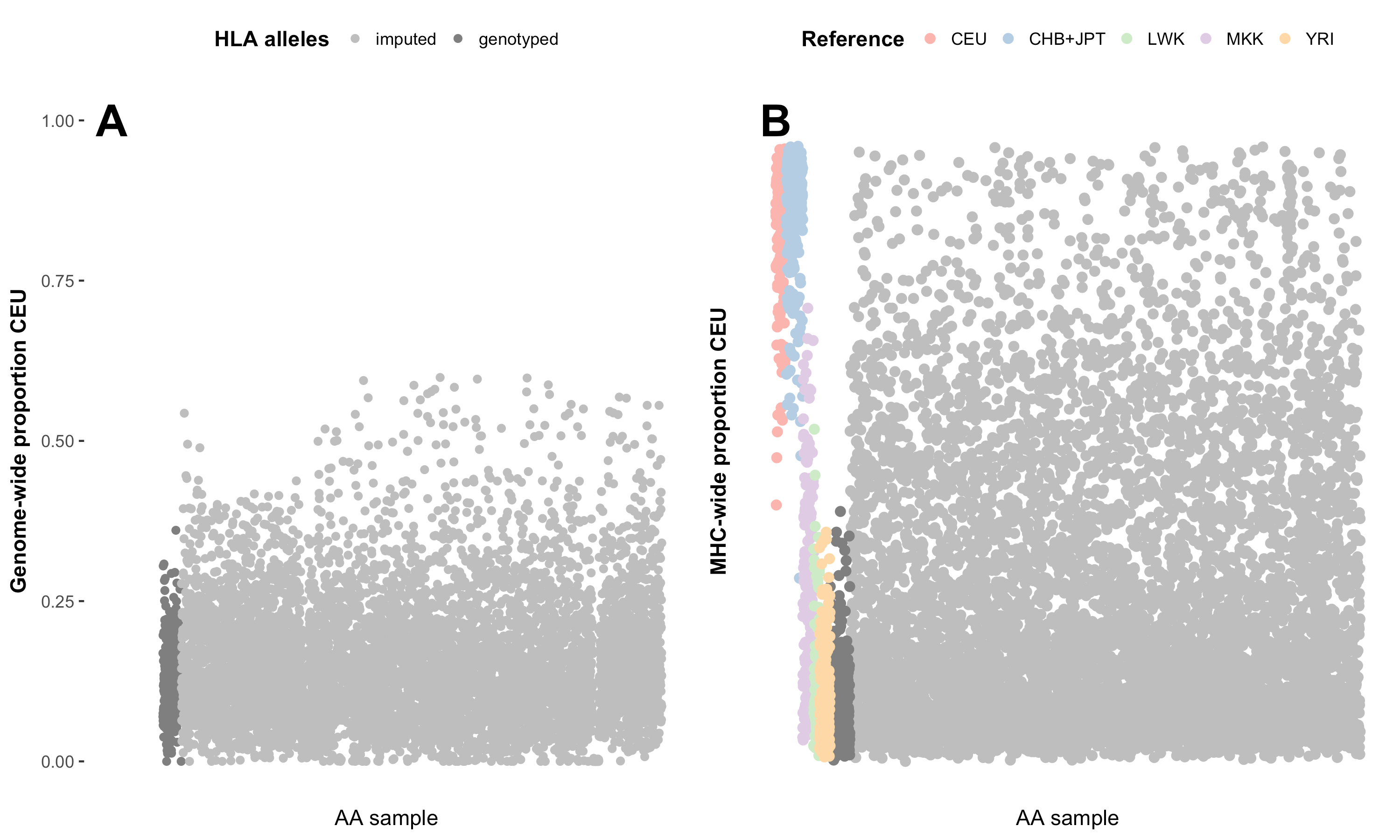
**

**Figure S5**

Admixture analysis. (A) Analysis of AA subjects on Genome Wide data. B) Analysis of AA subjects combined with hapmap subjects on MHC data. The AA subjects with HLA typing are in bold with the largest value at 0.4 well within the African Hapmap cluster.

| **Locus** | **N** | **Called** | **CallRate** | **Accuracy** |
| --- | --- | --- | --- | --- |
| ***A*** | 4 | 4 | 1 | 0.5 |
| ***B*** | 22 | 22 | 1 | 0.77 |
| ***C*** | 2 | 2 | 1 | 1 |
| ***DQA*** | 216 | 216 | 1 | 0.95 |
| ***DQB*** | 215 | 215 | 1 | 0.95 |
| ***DRB*** | 230 | 230 | 1 | 0.89 |

**Table S1: Cross validation results for the ‘African’ population reference data in HLA*IMPV2. The software predicts the HLA alleles for 1/3 of the reference data using the other 2/3 as training. The data in this table summarises the accuracy for these 1/3 test set.**

| **HLA ALLELE** | **R2** | **Sensitivity** | **Specificity** | **Frequency (N) Typed** | **Frequency (N) Imputed** |
| --- | --- | --- | --- | --- | --- |
| **DQA*01:01** | 0.877 | 1.000 | 0.991 | 0.068 (42) | 0.076 (47) |
| **DQA*01:02** | 0.881 | 0.959 | 0.980 | 0.360 (222) | 0.359 (221) |
| **DQA*01:03** | 0.595 | 0.778 | 0.997 | 0.015 (9) | 0.015 (9) |
| **DQA*02:01** | 0.844 | 1.000 | 0.990 | 0.062 (38) | 0.071 (44) |
| **DQA*03:01** | 0.480 | 0.500 | 0.998 | 0.036 (22) | 0.019 (12) |
| **DQA*04:01** | 0.974 | 0.986 | 0.998 | 0.229 (141) | 0.227 (140) |
| **DQA*05:01** | 0.947 | 0.993 | 0.990 | 0.224 (138) | 0.231 (142) |
| **DQA*05:02** | NA | 0.000 | 1.000 | 0.005 (3) | 0.000 (0) |
| **DQA*05:05** | NA | NA | 0.998 | 0.000 (0) | 0.002 (1) |
| **DQA*06:01** | NA | 0.000 | 1.000 | 0.002 (1) | 0.000 (0) |

**Table S2: Accuracy for HLA-DQA imputation in the 308 subjects used in our association study that were also typed (and included in the HLA*IMP-V2 reference set)**

| **HLA ALLELE** | **R2** | **Sensitivity** | **Specificity** | **Frequency Typed** | **Frequency Imputed** |
| --- | --- | --- | --- | --- | --- |
| **DQB*06:02** | 0.947 | 0.994 | 0.989 | 0.263 | 0.269 |
| **DQB*03:19** | 0.835 | 0.897 | 0.994 | 0.190 | 0.175 |
| **DQB*04:02** | 0.973 | 0.988 | 0.998 | 0.135 | 0.135 |
| **DQB*05:01** | 0.979 | 1.000 | 0.998 | 0.091 | 0.093 |
| **DQB*02:02** | 0.642 | 0.673 | 1.000 | 0.089 | 0.060 |
| **DQB*03:01** | 0.502 | 0.929 | 0.955 | 0.068 | 0.106 |
| **DQB*02:01** | 0.900 | 1.000 | 0.993 | 0.060 | 0.067 |
| **DQB*06:09** | 0.845 | 0.889 | 0.998 | 0.029 | 0.028 |
| **DQB*05:02** | 0.925 | 1.000 | 0.998 | 0.021 | 0.023 |
| **DQB*06:04** | 0.703 | 0.800 | 0.998 | 0.016 | 0.015 |
| **DQB*06:03** | 0.215 | 0.600 | 0.992 | 0.008 | 0.013 |
| **DQB*03:02** | 1.000 | 1.000 | 1.000 | 0.005 | 0.005 |
| **DQB*05:03** | 1.000 | 1.000 | 1.000 | 0.005 | 0.005 |
| **DQB*06:08** | NA | 0.000 | 1.000 | 0.005 | 0.000 |
| **DQB*02:03** | 1.000 | 1.000 | 1.000 | 0.003 | 0.003 |
| **DQB*03:03** | 0.664 | 1.000 | 0.998 | 0.003 | 0.005 |
| **DQB*03:09** | NA | 0.000 | 1.000 | 0.002 | 0.000 |
| **DQB*04:04** | NA | 0.000 | 1.000 | 0.002 | 0.000 |
| **DQB*06:39** | NA | 0.000 | 1.000 | 0.002 | 0.000 |

**Table S3: Accuracy for HLA-DQB imputation in the 308 subjects used in our association study that were also typed (and included in the HLA*IMP-V2 reference set)**

| **HLA ALLELE** | **R2** | **Sensitivity** | **Specificity** | **Frequency Typed** | **Frequency Imputed** |
| --- | --- | --- | --- | --- | --- |
| **DRB*15:03** | 0.970 | 0.978 | 1.000 | 0.222 | 0.218 |
| **DRB*03:02** | 0.947 | 0.953 | 1.000 | 0.138 | 0.131 |
| **DRB*08:04** | 0.967 | 0.972 | 1.000 | 0.115 | 0.112 |
| **DRB*11:01** | 0.821 | 1.000 | 0.984 | 0.073 | 0.088 |
| **DRB*11:02** | 0.604 | 0.854 | 0.976 | 0.067 | 0.080 |
| **DRB*03:01** | 0.894 | 0.972 | 0.995 | 0.058 | 0.062 |
| **DRB*07:01** | 0.794 | 1.000 | 0.986 | 0.058 | 0.071 |
| **DRB*13:02** | 0.792 | 0.971 | 0.988 | 0.057 | 0.067 |
| **DRB*13:03** | 0.725 | 0.783 | 0.998 | 0.037 | 0.031 |
| **DRB*01:02** | 1.000 | 1.000 | 1.000 | 0.034 | 0.034 |
| **DRB*13:01** | 0.442 | 0.563 | 0.997 | 0.026 | 0.018 |
| **DRB*13:04** | NA | 0.000 | 1.000 | 0.026 | 0.000 |
| **DRB*12:01** | 0.740 | 1.000 | 0.993 | 0.019 | 0.026 |
| **DRB*09:01** | 0.037 | 0.125 | 0.997 | 0.013 | 0.005 |
| **DRB*08:06** | 0.831 | 0.833 | 1.000 | 0.010 | 0.008 |
| **DRB*16:02** | 0.689 | 0.833 | 0.998 | 0.010 | 0.010 |
| **DRB*10:01** | 1.000 | 1.000 | 1.000 | 0.006 | 0.006 |
| **DRB*14:01** | 0.797 | 1.000 | 0.998 | 0.006 | 0.008 |
| **DRB*01:01** | 0.664 | 0.667 | 1.000 | 0.005 | 0.003 |
| **DRB*12:02** | 0.331 | 0.333 | 1.000 | 0.005 | 0.002 |
| **DRB*04:01** | 0.000 | 0.000 | 0.987 | 0.002 | 0.013 |
| **DRB*04:03** | NA | 0.000 | 1.000 | 0.002 | 0.000 |
| **DRB*04:05** | NA | 0.000 | 1.000 | 0.002 | 0.000 |
| **DRB*11:04** | NA | 0.000 | 1.000 | 0.002 | 0.000 |
| **DRB*11:10** | NA | 0.000 | 1.000 | 0.002 | 0.000 |
| **DRB*13:16** | NA | 0.000 | 1.000 | 0.002 | 0.000 |
| **DRB*13:37** | NA | 0.000 | 1.000 | 0.002 | 0.000 |
| **DRB*04:02** | NA | NA | 1.000 | 0.000 | 0.000 |
| **DRB*04:04** | NA | NA | 0.998 | 0.000 | 0.002 |
| **DRB*15:01** | NA | NA | 0.995 | 0.000 | 0.005 |
| **DRB*16:01** | NA | NA | 0.998 | 0.000 | 0.002 |

**Table S4: Accuracy for HLA-DRB1 imputation in the 308 subjects used in our association study that were also typed (and included in the HLA*IMP-V2 reference set)**

**Extended class II haplotypes**

*Most significant class II haplotype in African American*

**Tables S5 and S6** shows a series of conditional haplotype-based association tests used to unravel the most significant class II haplotype in AA and AA_sub_ *DRB*15:03—DQA*01:02—DQB*06:02* (p = 7.18x10^-22^, OR = 1.74). **Table S7** shows the top AA haplotype in EUR (p = 8.23x10^-10^, OR = 1.30).

**Table S5. African American subset**: Dissecting HLA class II haplotype associations

|  |  | *Nested models* | |  |  |
| --- | --- | --- | --- | --- | --- |
|  |  | **Models 2 and 4** | **Model 3 and 4** |  |  |
|  | **Model** | Δ -2lnL (p) | Δ -2lnL (p) | **BIC** | **AIC** |
|  |  |  |  |  |  |
|  | **DQA—DQB + DRB** |  |  |  |  |
| 1 | DRB*15:03—DQA*01:02—DQB*06:02 |  |  | 6763 | 6736 |
| 2 | DQA*01:02—DQB*06:02 | * |  | 6803 | 6775 |
| 3 | DRB*15:03 |  | * | 6758 | 6731 |
| 4 | DQA*01:02—DQB*06:02 + DRB*15:03 | 45.69 (1.39x10^-11^) | 1.12 (2.90x10^-01^) | 6766 | 6732 |
|  |  |  |  |  |  |
|  | **DRB—DQA + DQB** |  |  |  |  |
| 2 | DRB*15:03—DQA*01:02 | * |  | 6761 | 6734 |
| 3 | DQB*06:02 |  | * | 6805 | 6778 |
| 4 | DRB*15:03—DQA*01:02 + DQB*06:02 | 0.93 (3.35x10^-01^) | 44.64 (2.38x10^-11^) | 6769 | 6735 |
|  |  |  |  |  |  |
|  | **DRB—DQB + DQA** |  |  |  |  |
| 2 | DRB*15:03—DQB*06:02 | * |  | 6763 | 6736 |
| 3 | DQA*01:02 |  | * | 6844 | 6816 |
| 4 | DRB*15:03—DQB*06:02 + DQA*01:02 | 11.53 (6.83x10^-04^) | 91.76 (2.20x10^-16^) | 6761 | 6727 |
|  |  |  |  |  |  |
|  | *Models of allele pairs* |  |  |  |  |
|  | DQA*01:02 + DQB*06:02 |  |  | 6805 | 6771 |
|  | DQA*01:02 + DRB*15:03 |  |  | 6754 | 6720 |
|  | DQB*06:02 + DRB*15:03 |  |  | 6766 | 6732 |
|  |  |  |  |  |  |
|  | **DRB*15 2-digit resolution** |  |  |  |  |
| 2 | DQA*01:02—DQB*06:02 | * |  | 6802 | 6775 |
| 3 | DRB*15 |  | * | 6758 | 6731 |
| 4 | DQA*01:02—DQB*06:02 + DRB*15 | 48.47 (3.36x10^-12^) | 4.04 (4.43x10^-02^) | 6763 | 6729 |
|  |  |  |  |  |  |

Note: Pink highlight = overall best model fit by AIC; Light grey = DRB*15 2-digit resolution (presence of any of DRB*15:01, DRB*15:02, or DRB*15:03).

**Table S6. African American full**: Dissecting top AA HLA class II haplotype in AA full sample

|  |  | *Nested models* | |  |  |
| --- | --- | --- | --- | --- | --- |
|  |  | **Models 2 and 4** | **Model 3 and 4** |  |  |
|  | **Model** | Δ -2lnL (p) | Δ -2lnL (p) | **BIC** | **AIC** |
|  |  |  |  |  |  |
|  | **DQA—DQB + DRB** |  |  |  |  |
| 1 | DRB*15:03—DQA*01:02—DQB*06:02 |  |  | 7369 | 7341 |
| 2 | DQA*01:02—DQB*06:02 | * |  | 7402 | 7374 |
| 3 | DRB*15:03 |  | * | 7363 | 7336 |
| 4 | DQA*01:02—DQB*06:02 + DRB*15:03 | 38.34 (5.92x10^-10^) | 0.02 (8.76x10^-01^) | 7372 | 7338 |
|  |  |  |  |  |  |
|  | **DRB—DQA + DQB** |  |  |  |  |
| 2 | DRB*15:03—DQA*01:02 | * |  | 7366 | 7338 |
| 3 | DQB*06:02 |  | * | 7404 | 7376 |
| 4 | DRB*15:03—DQA*01:02 + DQB*06:02 | 0.02 (8.90x10^-01^) | 38.49 (5.59x10^-10^) | 7375 | 7340 |
|  |  |  |  |  |  |
|  | **DRB—DQB + DQA** |  |  |  |  |
| 2 | DRB*15:03—DQB*06:02 | * |  | 7369 | 7341 |
| 3 | DQA*01:02 |  | * | 7447 | 7419 |
| 4 | DRB*15:03—DQB*06:02 + DQA*01:02 | 5.37 (2.04x10^-02^) | 83.34 (2.20x10^-16^) | 7373 | 7338 |
|  |  |  |  |  |  |
|  | *Models of allele pairs* |  |  |  |  |
|  | DQA*01:02 + DQB*06:02 |  |  | 7407 | 7372 |
|  | DQA*01:02 + DRB*15:03 |  |  | 7366 | 7331 |
|  | DQB*06:02 + DRB*15:03 |  |  | 7372 | 7338 |
|  |  |  |  |  |  |
|  | **DRB*15 2-digit resolution** |  |  |  |  |
| *1* | DRB*15:03—DQA*01:02—DQB*06:02 |  |  | 7369 | 7341 |
| *2* | DQA*01:02—DQB*06:02 | * |  | 7402 | 7374 |
| *3* | DRB*15 |  | * | 7355 | 7327 |
| *4* | DQA*01:02—DQB*06:02 + DRB*15 | 50.51 (1.19x10^-12^) | 3.73 (5.34x10^-02^) | 7360 | 7325 |
|  |  |  |  |  |  |
|  | DRB*15:03—DQA*01:02 |  |  | 7366 | 7338 |
|  | DQA*01:02 + DRB*15:03 |  |  | 7366 | 7331 |
|  | DQA*01:02 + DRB*15:03 + 15:02 + 15:01 |  |  | 7356 | 7315 |
|  | DQA*01:02 + DRB*15 |  |  | 7349 | 7314 |

Note: Pink highlight = overall best model fit by AIC; Light grey = DRB*15 2-digit resolution (presence of any of DRB*15:01, DRB*15:02, or DRB*15:03); Green highlight = best fit model for the full AA sample (N=7402)

**Table S7. European**: Dissecting top AA HLA class II haplotype in EUR

|  |  | *Nested models* | |  |  |
| --- | --- | --- | --- | --- | --- |
|  |  | **Models 2 and 4** | **Model 3 and 4** |  |  |
|  | **Model** | Δ -2lnL (p) | Δ -2lnL (p) | **BIC** | **AIC** |
|  |  |  |  |  |  |
|  | **DQA—DQB + DRB** |  |  |  |  |
| 1 | DRB*15:01—DQA*01:02—DQB*06:02 |  |  | 14022 | 13978 |
| 2 | DQA*01:02—DQB*06:02 | * |  | 14018 | 13974 |
| 3 | DRB*15:01 |  | * | 14016 | 13972 |
| 4 | DQA*01:02—DQB*06:02 + DRB*15:01 | 2.00 (1.57x10^-01^) | <0.01(9.96x10^-01^) | 14025 | 13974 |
|  |  |  |  |  |  |
|  | **DRB—DQA + DQB** |  |  |  |  |
| 2 | DRB*15:01—DQA*01:02 | * |  | 14016 | 13972 |
| 3 | DQB*06:02 |  | * | 14018 | 13974 |
| 4 | DRB*15:01—DQA*01:02 + DQB*06:02 | <0.01 (9.67x10^-01^) | 2.28 (1.31x10^-01^) | 14025 | 13974 |
|  |  |  |  |  |  |
|  | **DRB—DQB + DQA** |  |  |  |  |
| 2 | DRB*15:01—DQB*06:02 | * |  | 14022 | 13978 |
| 3 | DQA*01:02 |  | * | 14014 | 13970 |
| 4 | DRB*15:01—DQB*06:02 + DQA*01:02 | 10.05 (1.53x10^-03^) | 2.17 (1.41x10^-01^) | 14021 | 13970 |
|  |  |  |  |  |  |
|  | *Models of allele pairs* |  |  |  |  |
|  | DQA*01:02 + DQB*06:02 |  |  | 14019 | 13968 |
|  | DQA*01:02 + DRB*15:01 |  |  | 14018 | 13967 |
|  | DQB*06:02 + DRB*15:01 |  |  | 14025 | 13974 |

Note: Pink highlight = overall best model fit by AIC

*Most significant class II haplotype in European*

**Table S8** show a series of conditional haplotype-based association tests used to unravel the most significant class II haplotype in EUR *DQA*05:01—DQB*02:01—DRB*03:01* (p = 2.58x10^-95^, OR = 2.32). **Tables S9** and **S10** shows the top EUR haplotype in AA and AA_sub_ (p = 3.42x10^-03^, OR = 1.27)

**Table S8. European**: Dissecting HLA class II haplotype associations

|  |  | *Nested models* | |  |  |
| --- | --- | --- | --- | --- | --- |
|  |  | **Models 2 and 4** | **Model 3 and 4** |  |  |
|  | **Model** | Δ -2lnL (p) | Δ -2lnL (p) | **BIC** | **AIC** |
|  |  |  |  |  |  |
|  | **DQA—DQB + DRB** |  |  |  |  |
| 1 | DRB*03:01—DQA*05:01—DQB*02:01 |  |  | 13617 | 13573 |
| 2 | DQA*05:01—DQB*02:01 | * |  | 13615 | 13572 |
| 3 | DRB*03:01 |  | * | 13623 | 13579 |
| 4 | DQA*05:01—DQB*02:01 + DRB*03:01 | 0.10 (7.51x10^-01^) | 7.44 (6.39x10^-03^) | 13625 | 13574 |
| *6* | *B*08:01* |  |  | 13628 | 13585 |
| *7* | *Model 1 + B*08:01* |  |  | 13558 | 13507 |
| *8* | *Model 2 + B*08:01* |  |  | 13557 | 13506 |
| *9* | *Model 3 + B*08:01* |  |  | 13562 | 13511 |
| *10* | *Model 4 + B*08:01* |  |  | 13566 | 13507 |
|  |  |  |  |  |  |
|  | **DRB—DQA + DQB** |  |  |  |  |
| 2 | DRB*03:01—DQA*05:01 | * |  | 13619 | 13575 |
| 3 | DQB*02:01 |  | * | 13614 | 13571 |
| 4 | DRB*03:01—DQA*05:01 + DQB*02:01 | 4.37 (3.67x10^-02^) | 0.07 (7.97x10^-01^) | 13624 | 13573 |
|  | *Model 2 + B*08:01* |  |  | 13559 | 13508 |
|  | *Model 3 + B*08:01* |  |  | 13556 | 13505 |
|  | *Model 4 + B*08:01* |  |  | 13565 | 13506 |
|  |  |  |  |  |  |
|  | **DRB—DQB+ DQA** |  |  |  |  |
| 2 | DRB*03:01—DQB*02:01 | * |  | 13616 | 13572 |
| 3 | DQA*05:01 |  | * | 13675 | 13631 |
| 4 | DRB*03:01—DQB*02:01 *+* DQA*05:01 | 0.96 (3.27x10^-01^) | 60.07 (9.16x10^-15^) | 13624 | 13573 |
|  | *Model 2 + B*08:01* |  |  | 13558 | 13507 |
|  | *Model 3 + B*08:01* |  |  | 13579 | 13528 |
|  | *Model 4 + B*08:01* |  |  | 13566 | 13508 |
|  |  |  |  |  |  |
|  | *Models of allele pairs* |  |  |  |  |
| 2 | DQA*05:01 + DQB*02:01 |  |  | 13622 | 13571 |
| 3 | DQA*05:01 + DRB*03:01 |  |  | 13632 | 13581 |
| 4 | DQB*02:01 + DRB*03:01 |  |  | 13624 | 13573 |
|  |  |  |  |  |  |

Note: Pink highlight = overall best model fit by AIC; Green highlight = best model fit by AIC, without B*08:01 class I allele

**Table S9. African American full**: Dissecting top EUR HLA class II haplotype in AA

|  |  | *Nested models* | |  |  |
| --- | --- | --- | --- | --- | --- |
|  |  | **Models 2 and 4** | **Model 3 and 4** |  |  |
|  | **Model** | Δ -2lnL (p) | Δ -2lnL (p) | **BIC** | **AIC** |
|  |  |  |  |  |  |
|  | **DQA—DQB + DRB** |  |  |  |  |
| 1 | DRB*03:01—DQA*05:01—DQB*02:01 |  |  | 7448 | 7420 |
| 2 | DQA*05:01—DQB*02:01 | * |  | 7447 | 7420 |
| 3 | DRB*03:01 |  | * | 7446 | 7418 |
| 4 | DQA*05:01—DQB*02:01 + DRB*03:01 | 1.62 (2.04x10^-01^) | <0.01 (9.52x10^-01^) | 7454 | 7420 |
|  | *B*08:01* |  |  | 7417 | 7390 |
|  | *Model 1 + B*08:01* |  |  | 7423 | 7389 |
|  | *Model 2 + B*08:01* |  |  | 7423 | 7389 |
|  | *Model 3 + B*08:01* |  |  | 7422 | 7388 |
|  | *Model 4 + B*08:01* |  |  | 7431 | 7389 |
|  |  |  |  |  |  |
|  | **DRB—DQA + DQB** |  |  |  |  |
| 2 | DRB*03:01—DQA*05:01 | * |  | 7446 | 7418 |
| 3 | DQB*02:01 |  | * | 7450 | 7422 |
| 4 | DRB*03:01—DQA*05:01 + DQB*02:01 | 0.18 (6.73x10^-01^) | 3.95 (4.69x10^-02^) | 7455 | 7420 |
|  | *Model 2 + B*08:01* |  |  | 7422 | 7388 |
|  | *Model 3 + B*08:01* |  |  | 7424 | 7390 |
|  | *Model 4 + B*08:01* |  |  | 7431 | 7389 |
|  |  |  |  |  |  |
|  | **DRB—DQB + DQA** |  |  |  |  |
| 2 | DRB*03:01—DQB*02:01 | * |  | 7448 | 7420 |
| 3 | DQA*05:01 |  | * | 7431 | 7403 |
| 4 | DRB*03:01—DQB*02:01 *+* DQA*05:01 | 17.61 (2.71x10^-05^) | 0.61 (4.35x10^-01^) | 7439 | 7404 |
|  | *Model 2 + B*08:01* |  |  | 7423 | 7389 |
|  | *Model 3 + B*08:01* |  |  | 7411 | 7377 |
|  | *Model 4 + B*08:01* |  |  | 7420 | 7379 |
|  |  |  |  |  |  |
|  | *Models of allele pairs* |  |  |  |  |
| 2 | DQA*05:01 + DQB*02:01 |  |  | 7439 | 7405 |
| 3 | DQA*05:01 + DRB*03:01 |  |  | 7438 | 7404 |
| 4 | DQB*02:01 + DRB*03:01 |  |  | 7454 | 7420 |
|  |  |  |  |  |  |

Note: Pink highlight = overall best model fit by AIC; Green highlight = best model fit by AIC, without B*08:01 class I allele

**Table S10. African American subset**: Dissecting top EUR HLA class II haplotype in AA

|  |  | *Nested models* | |  |  |
| --- | --- | --- | --- | --- | --- |
|  |  | **Models 2 and 4** | **Model 3 and 4** |  |  |
|  | **Model** | Δ -2lnL (p) | Δ -2lnL (p) | **BIC** | **AIC** |
|  |  |  |  |  |  |
|  | **DQA—DQB + DRB** |  |  |  |  |
| 1 | DRB*03:01—DQA*05:01—DQB*02:01 |  |  | 6844 | 6816 |
| 2 | DQA*05:01—DQB*02:01 | * |  | 6843 | 6816 |
| 3 | DRB*03:01 |  | * | 6844 | 6816 |
| 4 | DQA*05:01—DQB*02:01 + DRB*03:01 | 0.01 (7.53x10^-01^) | 0.44 (5.09x19^-01^) | 6852 | 6818 |
|  | *B*08:01* |  |  | 6821 | 6794 |
|  | *Model 1 + B*08:01* |  |  | 6828 | 6794 |
|  | *Model 2 + B*08:01* |  |  | 6828 | 6794 |
|  | *Model 3 + B*08:01* |  |  | 6828 | 6794 |
|  | *Model 4 + B*08:01* |  |  | 6836 | 6795 |
|  |  |  |  |  |  |
|  | **DRB—DQA + DQB** |  |  |  |  |
| 2 | DRB*03:01—DQA*05:01 | * |  | 6843 | 6816 |
| 3 | DQB*02:01 |  | * | 6845 | 6818 |
| 4 | DRB*03:01—DQA*05:01 + DQB*02:01 | 0.06 (8.00x10^-01^) | 2.16 (1.42x10^-01^) | 6852 | 6818 |
|  | *Model 2 + B*08:01* |  |  | 6827 | 6793 |
|  | *Model 3 + B*08:01* |  |  | 6828 | 6794 |
|  | *Model 4 + B*08:01* |  |  | 6836 | 6795 |
|  |  |  |  |  |  |
|  | **DRB—DQB + DQA** |  |  |  |  |
| 2 | DRB*03:01—DQB*02:01 | * |  | 6844 | 6816 |
| 3 | DQA*05:01 |  | * | 6829 | 6802 |
| 4 | DRB*03:01—DQB*02:01 *+* DQA*05:01 | 14.50 (1.40x10^-04^) | 0.18 (6.69x10^-01^) | 6838 | 6804 |
|  | *Model 2 + B*08:01* |  |  | 6828 | 6794 |
|  | *Model 3 + B*08:01* |  |  | 6828 | 6783 |
|  | *Model 4 + B*08:01* |  |  | 6826 | 6785 |
|  |  |  |  |  |  |
|  | *Models of allele pairs* |  |  |  |  |
| 2 | DQA*05:01 + DQB*02:01 |  |  | 6838 | 6804 |
| 3 | DQA*05:01 + DRB*03:01 |  |  | 6838 | 6804 |
| 4 | DQB*02:01 + DRB*03:01 |  |  | 6852 | 6818 |
|  |  |  |  |  |  |

Note: Pink highlight = overall best model fit by AIC; Green highlight = best model fit by AIC, without B*08:01 class I allele
